# Plastome sequences fail to resolve shallow level relationships within the rapidly radiated genus *Isodon* (Lamiaceae)

**DOI:** 10.3389/fpls.2022.985488

**Published:** 2022-09-08

**Authors:** Ya-Ping Chen, Fei Zhao, Alan J. Paton, Purayidathkandy Sunojkumar, Lian-Ming Gao, Chun-Lei Xiang

**Affiliations:** ^1^CAS Key Laboratory for Plant Diversity and Biogeography of East Asia, Kunming Institute of Botany, Chinese Academy of Sciences, Kunming, China; ^2^Royal Botanic Gardens, Kew, Richmond, United Kingdom; ^3^Department of Botany, University of Calicut, Thenhipalam, Kerala, India; ^4^Lijiang Forest Biodiversity National Observation and Research Station, Kunming Institute of Botany, Chinese Academy of Sciences, Lijiang, China

**Keywords:** genome skimming, Hengduan Mountains, Isodoninae, nutlet, plastid capture

## Abstract

As one of the largest genera of Lamiaceae and of great medicinal importance, *Isodon* is also phylogenetically and taxonomically recalcitrant largely ascribed to its recent rapid radiation in the Hengduan Mountains. Previous molecular phylogenetic studies using limited loci have only successfully resolved the backbone topology of the genus, but the interspecific relationships suffered from low resolution, especially within the largest clade (Clade IV) which comprises over 80% species. In this study, we attempted to further elucidate the phylogenetic relationships within *Isodon* especially Clade IV using plastome sequences with a broad taxon sampling of ca. 80% species of the genus. To reduce systematic errors, twelve different plastome data sets (coding and non-coding regions with ambiguously aligned regions and saturated loci removed or not) were employed to reconstruct phylogeny using maximum likelihood and Bayesian inference. Our results revealed largely congruent topologies of the 12 data sets and recovered major lineages of *Isodon* consistent with previous studies, but several incongruences are also found among these data sets and among single plastid loci. Most of the shallow nodes within Clade IV were resolved with high support but extremely short branch lengths in plastid trees, and showed tremendous conflicts with the nrDNA tree, morphology and geographic distribution. These incongruences may largely result from stochasticity (due to insufficient phylogenetic signal) and hybridization and plastid capture. Therefore, the uniparental-inherited plastome sequences are insufficient to disentangle relationships within a genus which has undergone recent rapid diversification. Our findings highlight a need for additional data from nuclear genome to resolve the relationships within Clade IV and more focused studies to assess the influences of multiple processes in the evolutionary history of *Isodon*. Nevertheless, the morphology of the shape and surface sculpture/indumentum of nutlets is of systematic importance that they can distinguish the four major clades of *Isodon*.

## Introduction

*Isodon* (Schrad. ex Benth.) Spach (Ocimeae, Nepetoideae, Lamiaceae) consists of approximately 100 species, mostly occurring in subtropical to tropical Asia, with two endemic species disjunctly distributed in tropical Africa (Li, 1988; Li and Hedge, 1994; Paton et al., 2009). It is most diverse in southwest China, particularly in the dry valleys of the global biodiversity hotspot Hengduan Mountains (HM), which is considered as the distribution center of the genus (Zhong et al., 2010; Yu et al., 2014; Chen et al., 2019). As a member of the monotypic subtribe Isodoninae established by Zhong et al. (2010), *Isodon* differs from other genera of Ocimeae by the following set of characters: perennial herbs, subshrubs or shrubs, pedunculate and bracteolate cymes, actinomorphic or two-lipped (3/2) calyces, strongly two-lipped (4/1) corollas, and free filaments inserted at the base of the corolla tubes (Wu and Li, 1977; Li, 1988; Paton and Ryding, 1998; Harley et al., 2004). In a worldwide revision of *Isodon*, Li (1988) divided the genus into four sections – sect. *Pyramidium* (Benth.) H.W. Li (7 spp.), sect. *Amethystoides* (Benth.) H.W. Li (7 spp.), sect. *Isodon* (91 spp.), and sect. *Melissoides* (Benth.) H.W. Li (6 spp.) – based on the types of inflorescence, and morphology of fruiting calyx (erect vs. decurved, actinomorphic vs. two-lipped) and corolla tube (saccate vs. gibbous to shortly calcarate on upper side near base). Ten series were further delimited within the largest section (sect. *Isodon*) by Li (1988).

Some species of *Isodon* [e.g., *I*. *eriocalyx* (Dunn) Kudô, *I*. *japonicus* (Burm. f.) H. Hara, *I*. *rubescens* (Hemsl.) H. Hara] have long been used as traditional folk medicine in China and Japan and the genus is abundant in diterpenoids with diverse structural scaffolds and important pharmaceutical functions (Sun et al., 2006; Liu et al., 2017). But despite its apparent value to medicine and understanding of the evolutionary history of the HM flora, several large gaps in our understanding of the taxonomy and systematics of *Isodon* remain and restrict our ability to effectively communicate its infrageneric taxonomic units.

*Isodon* species are well-known for being difficult to identify, with either diagnostic characters too variable and/or obscure, or having been provided with incomplete descriptions due to a lack of sufficient specimens and/or field investigations (Chen et al., 2019). Low phylogenetic resolution of the infrageneric relationships within *Isodon* from previous molecular phylogenetic studies (Zhong et al., 2010; Yu et al., 2014; Chen et al., 2019) also hampers our ability to assemble a comprehensive and intelligible taxonomy for the genus. Using limited molecular markers, all these studies consistently revealed that Asian *Isodon* can be divided into three strongly supported clades and the relationships within the largest clade which includes more than 80% species of the genus were scarcely resolved with weak to non-existent support. The analyses by Yu et al. (2014) also provided support for a clade of two endemic African species sister to the combination of three Asian clades, whose exact relationship with each other was recovered as equivocal. Moreover, these studies showed that at least three of the aforementioned four sections of *Isodon* proposed by Li (1988) based on the inflorescence types and fruiting calyx and corolla tube morphology were not monophyletic, whereas the color of glands on plants and leaf phyllotaxy might be of systematic significance (Zhong et al., 2010; Yu et al., 2014). The biogeographic study of *Isodon* (Yu et al., 2014) indicated that the genus originated in the Qinghai-Tibetan Plateau (QTP) and adjacent regions in the late Oligocene, and a rapid radiation of the genus triggered by the uplift of QTP and subsequent aridification events might have happened in the late Miocene. They also concluded that the rapid diversification followed by hybridization and introgression might have resulted in the greatest diversity of *Isodon* in HM and the low phylogenetic resolution within the genus (Yu et al., 2014).

Due to the low evolutionary rates, high copy number, uniparental inheritance, lack of recombination or gene duplication compared to the nuclear genome (Birky, 1995; Wicke et al., 2011; Gitzendanner et al., 2018), plastid genomes (plastomes) have been widely used for phylogenetic reconstruction at various levels during the last decade (Yu et al., 2017; Fu et al., 2019; Li et al., 2019, 2021; Xiang et al., 2020; Zhao et al., 2021b; Wang et al., 2022). Meanwhile, an increasing number of studies have demonstrated that substantial improvements in phylogenetic resolution within rapidly radiated genera can be achieved using plastome-scale data sets (e.g., Foster et al., 2018; Ji et al., 2019; Zong et al., 2019; He et al., 2021; Zhao et al., 2021a; Cao et al., 2022). However, most of these infrageneric plastid phylogenomic studies were based on limited taxon sampling, which may reduce the accuracy of phylogenetic inference (Graybeal, 1998; Hillis, 1998; Zwickl and Hillis, 2002; Heath et al., 2008).

In this study, we carried out the phylogenetic analyses of 86 taxa of *Isodon* using complete plastome and nuclear ribosomal DNA [nrDNA, including 26S, 18S, and 5.8S ribosomal RNA genes, and internal and external transcribed spacers (ITS and ETS)] sequences, representing the most comprehensive taxonomic sampling of the genus to date. We aimed to answer the following questions: (a) whether plastome-scale data can further resolve the shallow-level relationships within *Isodon* based on a comprehensive taxa sampling? (b) if there are any conflicts among different plastome data sets, among single plastid genes, and between the plastid and nrDNA data sets? What might be the causes of these incongruences?

## Materials and methods

### Taxon sampling

A total of 99 accessions of 86 taxa (including 80 species and six varieties) from the major distribution areas of *Isodon* in East Asia and Africa were sampled as ingroups, representing all recognized sections and series of Li (1988) and all four clades recovered in Yu et al. (2014). Sixteen species from 11 genera of the other six subtribes of Ocimeae were selected as outgroups. Plastomes of 106 individuals were newly generated here from genome skimming data, plus nine plastomes downloaded from GenBank. The sequences of 18S, ITS1, 5.8S, ITS2, and 26S (hereafter referred to as nrITS) were mostly assembled from the genome skimming data, with several sequences obtained by polymerase chain reaction (PCR) amplification or downloaded from GenBank. The ETS sequences generated from the same collections of about half of the species have been published before (Chen et al., 2019), thus these sequences were downloaded from the GenBank. The remaining ETS sequences were newly obtained from PCR amplification. Voucher information and GenBank accession numbers for all sequences are provided in Supplementary Table 1. Vouchers of most accessions were deposited at the Herbarium of Kunming Institute of Botany (KUN), Chinese Academy of Sciences.

### DNA extraction, amplification, and sequencing

Total genomic DNA was either extracted from silica-gel dried leaves using a modified CTAB method (Doyle and Doyle, 1987) or from herbarium specimens using the DNeasy Plant Mini Kit (Tiangen Biotech, Beijing, China) according to the manufacturer’s instructions. Genomic DNA was then sheared into ca. 300 bp fragments, which were used for library construction following standard protocols (NEBNext^®^ Ultra IITMDNA Library Prep Kit for Illumina^®^). Sequencing with 2 × 150 bp paired-end reads was conducted to generate approximately 2 Gb data for each accession using an Illumina HiSeq 2000 platform (Illumina, San Diego, CA, United States) at BGI Genomics (Shenzhen, Guangdong, China).

PCR mixtures and procedures for the amplification of ITS (including partial 18S, ITS1, 5.8S, ITS2, and partial 26S) and ETS followed those described in Chen et al. (2016a), using primer pairs of 17SE and 26SE (Sun et al., 1994), and ETS-B (Beardsley and Olmstead, 2002) and 18S-IGS (Baldwin and Markos, 1998) for ITS and ETS, respectively. The PCR products were purified and sequenced by the Sangon Biotech (Shanghai, China) on an ABI 3730xl DNA Analyzer (Applied Biosystems, CA, United States).

### Plastome and nrDNA assembly and annotation

Adaptors and low-quality reads were removed from the raw data using Trimmomatic v.0.32 (Bolger et al., 2014) with default settings. Subsequently, the *de novo* assembling of clean paired-end reads was carried out using the GetOrganelle Toolkit (Jin et al., 2020), and the resulting contigs were further visualized and edited using Bandage v.0.8.1 (Wick et al., 2015). The newly assembled plastomes were initially annotated with the Plastid Genome Annotator (PGA) (Qu et al., 2019). Using the published plastome of *Isodon amethystoides* (Benth.) H. Hara (GenBank accession number: MT473767; Zhao et al., 2021b) as a reference, the start and stop codons and intron/exon boundaries for protein-coding genes were checked manually in Geneious v.11.0.3 (Kearse et al., 2012). Annotated tRNA genes were verified using the online tRNAscan-SE service (Chan and Lowe, 2019).

The nrITS sequences were *de novo* assembled from the clean data using the GetOrganelle Toolkit (Jin et al., 2020) and the annotation was carried out in Geneious by comparison with the reference sequence of *Perilla frutescens* (L.) Britton (GenBank accession number: KT220698; Cheon et al., 2018). For the sequences of ITS and ETS resulted from Sanger sequencing, trace files with both directions were assembled and edited using Geneious.

### Sequence alignment and data set construction

The script “get_annotated_regions_from_gb.py” developed by Zhang et al. (2020) was employed to extract coding and non-coding regions from whole plastomes with one of the inverted repeats (IR) removed. Alignment of individual loci was performed with MAFFT v.7.4.0 (Katoh and Standley, 2013) using the L-INS-i algorithm and manually adjusted in MEGA 6.0 (Tamura et al., 2013). To minimize the use of loci with limited information, aligned regions less than 25 bp and the conserved rRNAs and tRNAs were excluded from analyses. A total of 207 alignments, including 80 coding genes and 127 non-coding loci, were obtained. The ITS and ETS sequences were aligned separately using the MAFFT plugin in Geneious and then manually adjusted in MEGA.

For the plastome sequences, a total of 12 data sets were generated prior to the phylogenetic reconstruction. Three basic data sets were produced initially: the CR (coding regions; the concatenated 80 coding genes), NCR (non-coding regions; the concatenated 127 non-coding loci), and CR + NCR (the concatenated CR and NCR) data sets. The script “concatenate_fasta.py” developed by Zhang et al. (2020) was used to concatenate the alignments of individual loci. To reduce the impact of misalignment, three data sets CR-GB, CR-GB, and CR + NCR-GB were constructed after using Gblocks v.0.91b (Castresana, 2000; Talavera and Castresana, 2007) to exclude ambiguously aligned regions with default parameters (“Allowed Gap Positions” = “With Half”). Loci with high levels of substitutional saturation were also identified and excluded to construct six data sets. The degree of saturation of all 207 loci was calculated using two indices determined by TreSpEx v.1.1 (Struck, 2014): the slope of the linear regression of plotting patristic distance against uncorrected *p* distances and the *R*^2^ fit of the data to this slope. The higher the slope and *R*^2^, the less saturated is the locus. The slope and *R*^2^ values of all loci are summarized in Supplementary Table 2, and the density plots of these values (Supplementary Figure 1) were generated using R v.4.0.5 (R Development Core Team, 2021). The threshold of the distribution of slopes for exclusion of loci was determined at the value of 0.85 (Supplementary Figure 1). Thus, three data sets (CR-Slope, NCR-Slope, CR + NCR-Slope) were produced by excluding the 72 loci (11 coding and 61 non-coding loci; Supplementary Table 2) with slopes lower than 0.85 (determined at which the distribution of the slope values increase significantly). The remaining three data sets (CR-*R*^2^, NCR-*R*^2^, CR + NCR-*R*^2^) were generated after removing the 19 loci (three coding and 16 non-coding loci; Supplementary Table 2) located on the left distribution of *R*^2^ (lower than the value of 0.90, at which the distribution of *R*^2^ began to increase; Supplementary Figure 1).

For the nuclear sequences, the nrITS and ETS regions were directly concatenated to construct the nrDNA data set.

### Phylogenetic analysis

Phylogenetic reconstruction was carried out using maximum likelihood (ML) and Bayesian inference (BI) analyses for each of the 12 plastome data sets and the nrDNA data set. The best partitioning schemes and substitution models for the 12 plastome data sets were estimated by PartitionFinder2 v.2.1.1 (Lanfear et al., 2017) with the selection of all model, RAxML v.8.2.12 (Stamatakis, 2014), the rcluster algorithm (Lanfear et al., 2014), and the corrected Akaike information criterion (AICc). The nrDNA data set was partitioned by the nrITS and ETS region, and the best substitution models for each DNA locus were selected under the AIC using jModelTest 2.1.6 (Darriba et al., 2012).

Partitioned ML analysis was implemented using RAxML-HPC2 v.8.2.12 (Stamatakis, 2014) on XSEDE on the web server Cyberinfrastructure for Phylogenetic Research Science (CIPRES) Gateway (Miller et al., 2010).^1^ Analysis of 1,000 rapid bootstrap replicates (-x) was followed by a search for the best-scoring ML tree in a single program run (-f a), both phases using the GTRGAMMA model for nucleotide data and other parameters being the default settings.

Partitioned BI analysis was performed with MrBayes v.3.2.7a (Ronquist et al., 2012) on XSEDE on the CIPRES Gateway. The Markov chain Monte Carlo (MCMC) analyses were run for 20,000,000 generations. Stationarity was considered to be reached when the average standard deviation of split frequencies (ASDSF) fell below 0.01. Trees were sampled at every 1,000 generations with the first 25% discarded as burn-in. The remaining trees were used to build a 50% majority-rule consensus tree.

Gene trees for each of the 207 loci extracted from plastome sequences were also reconstructed using RAxML-HPC2 for 1,000 rapid bootstrap replicates and a search for the best-scoring tree.

### Analyses of topological concordance

To explore the concordance/discordance among the 12 data sets of plastome, concatenated gene trees inferred by RAxML from each of the 12 data sets was iteratively used as a reference tree for mapping the remaining trees. The analyses were implemented using PhyParts (Smith et al., 2015) with all trees rooted. To minimize the effect of gene tree estimation error, bipartitions with bootstrap values lower than 70% were ignored for congruence calculations. Results were visualized using the ETE3 Python toolkit (Huerta-Cepas et al., 2016) as implemented in PhyParts_PieCharts.^2^ Concordance among single gene trees was also analyzed using PhyParts by mapping the RAxML trees inferred from the 207 loci against the CR + NCR-GB tree, which is the best resolved and most concordant with the remaining 11 concatenated trees. All 207 single gene trees were rooted and only bipartitions with at least 70% BS were considered informative. Finally, incongruence between the CR + NCR-GB tree and the nrDNA tree was visualized using the “tanglegram” function in Dendroscope v.3.8.2 (Huson and Scornavacca, 2012). To minimize spurious disagreement between the two trees due to estimation error, all bipartitions with lower than 50% BS were collapsed.

### Morphological and geographical data collection

To test the congruence between morphological and molecular data within *Isodon*, morphological similarities and differences were analyzed based on our previous field investigations and specimen examination. Specimens of *Isodon* and related genera from 29 herbaria (A, AU, BM, CDBI, CSFI, E, G, GXMI, HHBG, HIB, IBK, IBSC, K, KUN, KYO, L, LBG, LE, MW, NAS, P, PE, S, SYS, SZ, TAI, TI, W, and WUK; abbreviations follow Thiers, 2022) were examined. The systematic significance of nutlet morphology was also investigated using light microscopy (LM) and scanning electron microscopy (SEM). Mature nutlets were collected from natural populations and herbarium collections. A total of 61 taxa of *Isodon* were selected (Supplementary Table 4). Except for some species where only 1–5 nutlets were available, most species were represented by 10–20 mature nutlets. Measurements and LM analysis were carried out using the Keyence VHX-6000 digital microscope (Keyence Corporation, Osaka, Japan). For SEM, nutlets were directly mounted onto stubs and sputter-coated with gold. Micromorphological observations were conducted using the Hitachi S4800 (Hitachi Ltd., Tokyo, Japan) or Zeiss EVO LS10 (Carl Zeiss NTS, Oberkochen, Germany) scanning electron microscopes at 10 kV. Terminologies used for nutlet description followed those of Budantsev and Lobova (1997) and Moon et al. (2009).

To test the congruence between geographical and molecular data within *Isodon*, the extant distribution data of each species was compiled from taxonomic and floristic literature (Wu and Li, 1977; Li, 1988; Murata and Yamazaki, 1993; Suddee et al., 2004; Paton et al., 2009) and herbarium records. Five biogeographic regions were delimited based on Yu et al. (2014): (A) QTP and adjacent regions; (B) tropical Asia; (C) eastern Asia; (D) Japan; (E) tropical Africa.

## Results

### Plastome features of *Isodon*

All *Isodon* plastomes newly assembled here displayed the typical quadripartite structure, including a large single copy (LSC) region and a small single copy (SSC) region separated by two inverted repeat (IRa and IRb) regions (Supplementary Figure 2). The complete plastomes ranged in length from 151,923 bp [*I. yuennanensis* (Hand.-Mazz.) H. Hara] to 152,824 bp [*I. ternifolius* (D. Don) Kudô] (Supplementary Figure 2 and Supplementary Table 3). The LSC regions ranged from 82,906 bp (*I. yuennanensis*) to 83,640 bp (*I. ternifolius*), the SSC regions varied between 17,532 bp [*I. megathyrsus* (Diels) H. Hara] and 17,729 bp [*I. lophanthoides* var. *graciliflorus* (Benth.) H. Hara], and the IR regions ranged from 25,671 bp [*I. phyllopodus* (Diels) Kudô] to 25,759 bp [*I. rugosus* (Wall. ex Benth.) Codd]. The overall GC content of the *Isodon* plastomes was similar and ranged from 37.5% in *I. ramosissimus* (Hook. f.) Codd to 37.7% in *I. pharicus* (Prain) Murata and *I. adenanthus* (Diels) Kudô (Supplementary Table 3). Plastomes of *Isodon* consist of a total of 114 unique genes, including 80 protein coding genes, 30 tRNA genes, and four rRNA genes, and the gene order was highly conserved (Supplementary Figure 2 and Supplementary Table 3).

### Phylogenetic relationships and conflicts among data sets

The sequence characteristics of all 12 plastome data sets and the nrDNA data set were summarized in Table 1. The alignment length of the nrDNA matrix was 6,371 bp, but with the highest percentage of parsimonious-informative sites (9.65%). Among the 12 plastome data sets, the alignment length of the CR + NCR data set (129,827 bp) was the longest, and that of the NCR-Slope data set was the shortest (28,319 bp), whereas the NCR-GB data set contained the highest percentage of variable sites (15.94%) and parsimonious-informative sites (7.82%).

**TABLE 1:** Detailed characteristics of all plastome data sets and the combined nrDNA data set used in present study.

Data set	No. of loci	No. of sites [bp]	No. of variable sites [bp]	No. of parsimonious-informative sites [bp]
CR	80	69,430	6,696 (9.64%)	3,357 (4.84%)
CR-GB	80	68,694	6,649 (9.68%)	3,339 (4.86%)
CR-Slope	69	64,327	6,275 (9.75%)	3,115 (4.84%)
CR-*R*^2^	77	67,762	6,561 (9.68%)	3,282 (4.84%)
NCR	127	60,397	8,833 (14.62%)	4,223 (6.99%)
NCR-GB	127	50,540	8,054 (15.94%)	3,950 (7.82%)
NCR-Slope	66	28,319	3,020 (10.66%)	1,432 (5.06%)
NCR-*R*^2^	111	55,486	8,077 (14.56%)	3,862 (6.96%)
CR + NCR	207	129,827	15,529 (11.96%)	7,580 (5.84%)
CR + NCR-GB	207	119,234	14,703 (12.33%)	7,289 (6.11%)
CR + NCR-Slope	135	92,646	9,295 (10.03%)	4,547 (4.91%)
CR + NCR-*R*^2^	188	123,248	14,638 (11.88%)	7,144 (5.80%)
nrDNA	2	6,371	936 (14.69%)	615 (9.65%)

All 12 plastome data sets yielded almost identical tree topologies at deep nodes and exhibited largely congruent relationships at shallow nodes with some differences or conflicts (Supplementary Figure 3). For the three data sets consisting of coding and/or non-coding regions and without further treatment, the phylogenetic tree resulted from the complete plastome (CR + NCR data set) had the highest resolution, followed by the NCR and the CR data sets. Several hard incongruences can be found between the NCR tree and the CR tree, whereas these conflicting nodes in the CR + NCR tree were either collapsed into polytomies or similar to the better supported ones. After removing sites that were poorly aligned, the phylogenetic relationships and support values in the CR + NCR-GB tree and the CR-GB tree were almost unchanged (even slightly better) compared with the tree topologies prior to the treatment. However, the resolution of the NCR-GB tree was slightly poorer than that of the NCR tree, as significantly larger number of misaligned sites pruned from the NCR data set than from the CR data set (Table 1). After excluding possible saturated loci with *R*^2^ values lower than 0.90 (three coding and 16 non-coding loci), the resolution of the CR-*R*^2^ tree was largely consistent with that of CR tree, but were slightly poorer in the NCR-*R*^2^ tree and CR + NCR-*R*^2^ tree compared with the NCR tree and CR + NCR tree, respectively. Likewise, the CR-slope tree was also consistent with the CR tree after removing eleven coding loci with slope values lower than 0.85. In contrast, with nearly half (61 out of 127) of the non-coding loci excluded, the NCR-slope tree had the lowest resolution among the 12 concatenated trees. Interestingly, the support value of the sister clade of *Isodon* formed by ten outgroup species was improved by ambiguously aligned regions and saturated loci being removed.

**FIGURE 1:**
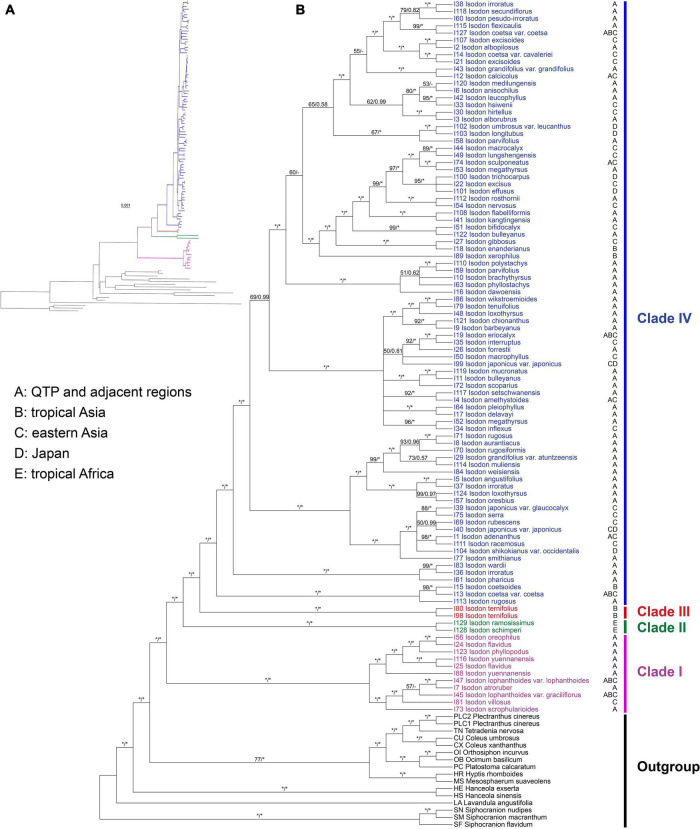
Cladogram **(A)** and phylogram **(B)** of the maximum-likelihood tree of *Isodon* derived from the plastid phylogenomic analysis of a concatenated data set including 80 coding and 127 non-coding loci with ambiguously aligned regions removed (CR + NCR-GB data set). Support values ≥ 50% BS or 0.50 PP are displayed above the branches (“*” indicates a support value = 100% BS or 1.00 PP, “–” indicates a support value < 0.50 PP). The distribution area(s) of each *Isodon* species is shown beside the tip and letters coding for areas follow Yu et al. (2014).

The ML tree resulted from the CR + NCR-GB data set was used as the main reference tree (Figure 1), as it was the best resolved and most concordant with the remaining 11 concatenated trees. The BI tree was also consistent with the ML tree of the same data set and among the 12 data sets, therefore, only PP support values of the BI tree were superimposed together with BS support values on the nodes of the ML tree (Figure 1). *Isodon* was shown to be monophyletic (BS = 100%/PP = 1.00) and four major clades (Clades I–IV) were recovered within the genus. As the first diverging clade, Clade I (BS = 100%/PP = 1.00) consisted of 11 taxa which can be further divided into two strongly supported subclades. The first subclade comprised *I. lophanthoides* (Buch.-Ham. ex D. Don) H. Hara var. *lophanthoides*, *I. lophanthoides* var. *graciliflorus*, *I. atroruber* R.A. Clement, *I. villosus* Y.P. Chen and H. Peng, and *I. scrophularioides* (Wall. ex Benth.) Murata, and the other subclade included *I. oreophilus* (Diels) A.J. Paton and Ryding, *I. flavidus* (Hand.-Mazz.) H. Hara. *I. phyllopodus*, and *I. yuennanensis*. The next split lineage was composed of the two African endemic species *I. ramosissimus* and *I. schimperi* (Vatke) J.K. Morton (Clade II; BS = 100%/PP = 1.00). Clade III consists of the two individuals of *I. ternifolius* (BS = 100%/PP = 1.00), which was further sister to the largest Clade IV (BS = 100%/PP = 1.00). Despite a few shallow nodes were collapsed into polytomies or weakly supported, most of the nodes within Clade IV were resolved with robust support. However, the internal branch lengths within Clade IV were extremely short. The single gene trees were largely unresolved, with some of the loci exhibiting conflicts with the concatenated data set (Supplementary Figure 4).

**FIGURE 2:**
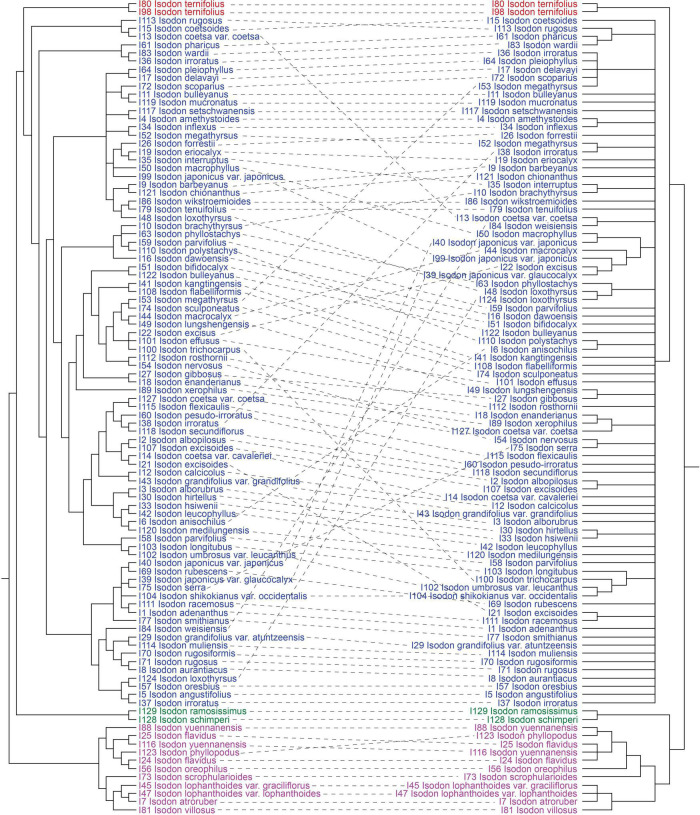
Tanglegram comparing plastid (maximum-likelihood tree inferred from the CR + NCR-GB data set) and nuclear (maximum-likelihood tree inferred from the nrDNA data set) trees, optimized in Dendroscope to minimize line crossings. All clades with < 50% BS have been collapsed.

Consistent with the plastid tree, the nrDNA tree (Supplementary Figure 5) also recovered *Isodon* as a monophyletic group (BS = 87%/PP = 1.00) consisting of four major lineages: Clade I (BS = 96%/PP = 1.00), Clade II (BS = 100%/PP = 1.00), Clade III (BS = 100%/PP = 1.00), and Clade IV (BS = 72%/PP = 1.00). Even though the relationships within Clade IV of the nrDNA tree were almost unresolved with a large polytomy, the plastid-nuclear tanglegram demonstrated widespread discordances (Figure 2). In contrast with the plastid tree, Clade II was shown to be sister to Clade I in the nrDNA tree (BS = 89%/PP = 1.00). The placement of *I. scrophularioides* within Clade I also varied between the two genomic data sets that it was sister to the subclade formed by *I. yuennanensis*, *I. flavidus*, *I. phyllopodus*, and *I. oreophilus* in the nuclear tree (BS = 61%/PP = 0.87; Supplementary Figure 5). Most of the incongruences were located within Clade IV, which comprised over 80% sampled taxa of *Isodon*.

### Nutlet morphology of *Isodon* species

**FIGURE 3:**
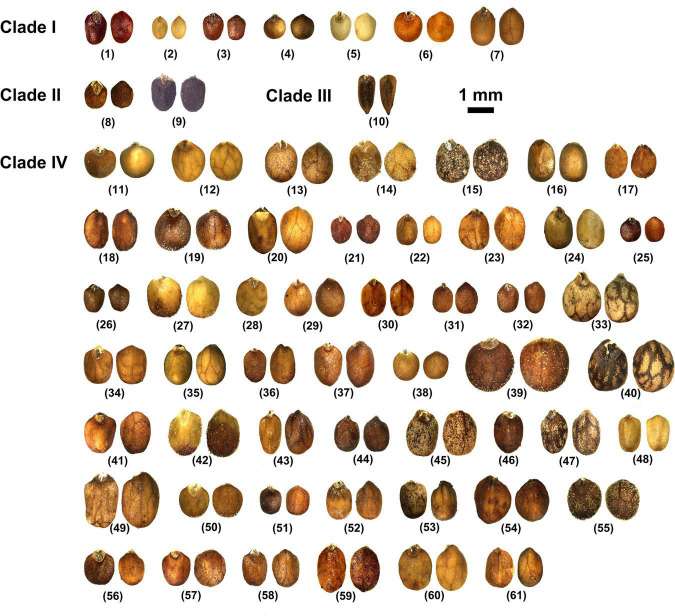
Nutlet morphology of taxa of *Isodon*. (1) *I. atroruber*; (2) *I. lophanthoides* var. *lophanthoides*; (3) *I. lophanthoides* var. *graciliflorus*; (4) *I. oreopilus*; (5) *I. phyllopodus*; (6) *I. scrophularioides*; (7) *I. villosus*; (8) *I. ramosissimus*; (9) *I. schimperi*; (10) *I. ternifolius*; (11) *I. adenanthus*; (12) *I. albopilosus*; (13) *I. alborubrus*; (14) *I. amethystoides*; (15) *I. angustifolius*; (16) *I. anisochilus*; (17) *I. aurantiacus*; (18) *I. barbeyanus*; (19) *I. bifidocalyx*; (20) *I. bulleyanus*; (21) *I. coetsa* var. *coetsa*; (22) *I. coetsa* var. *cavaleriei*; (23) *I. dawoensis*; (24) *I. delavayi*; (25) *I. enanderianus*; (26) *I. eriocalyx*; (27) *I. excisus*; (28) *I. forrestii*; (29) *I. gibbosus*; (30) *I. grandifolius* var. *atuntzeensis*; (31) *I. hirtellus*; (32) *I. interruptus*; (33) *I. irroratus*; (34) *I. japonicus* var. *glaucocalyx*; (35) *I. kangtingensis*; (36) *I. leucophyllus*; (37) *I. loxothyrsus*; (38) *I. lungshengensis*; (39) *I. macrocalyx*; (40) *I. macrophyllus*; (41) *I. megathyrsus*; (42) *I. nervosus*; (43) *I. oresbius*; (44) *I. parvifolius*; (45) *I. pharicus*; (46) *I. phyllostachys*; (47) *I. pleiophyllus*; (48) *I. polystachys*; (49) *I. pseudoirroratus*; (50) *I. rosthornii*; (51) *I. rubescens*; (52) *I. rugosu*s; (53) *I. scoparius*; (54) *I. sculponeatus*; (55) *I. serra*; (56) *I. setchwanensis*; (57) *I. smithianus*; (58) *I. ternuifolius*; (59) *I. wardii*; (60) *I. weisiensis*; (61) *I. wikstroemioides*.

**FIGURE 4:**
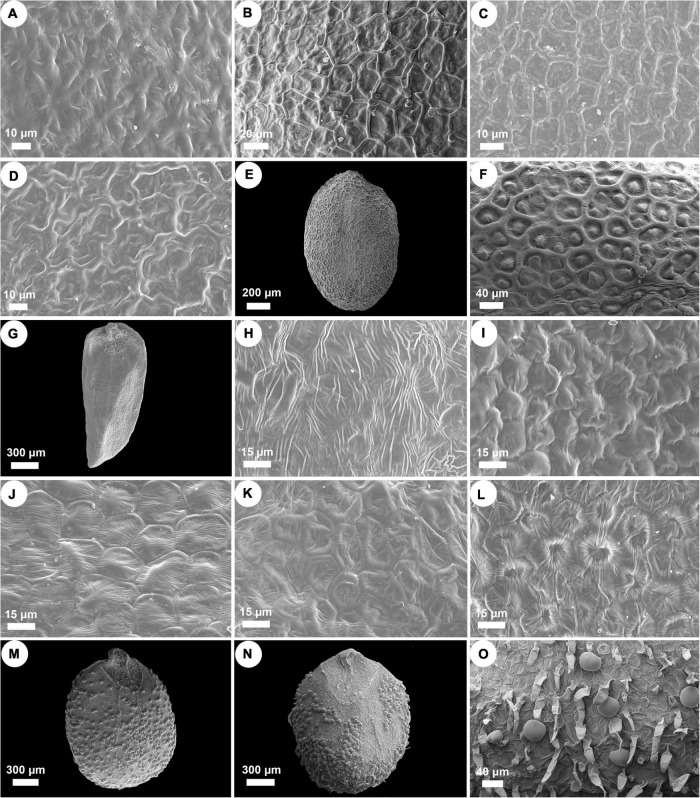
SEM micrographs of nutlet surface pattern in *Isodon*. **(A)** Psilate surface. *I. lophanthoides*. **(B–D)** Reticulate surface. **(B)** *I. oreophilus*; **(C)** *I. phyllopodus*; **(D)** *I. scrophularioides*. **(E,F)** Reticulate-papillate surface. *I. schimperi*. **(G,H)** Striate surface. *I. ternifolius*. **(I–L)** Cellular surface. **(I)** *I. barbeyanus*; **(J)** *I. dawoensis*; **(K)** *I. scoparius*; **(L)** *I. smithianus*. **(M–O)** Glandular or glandular and pubescent surface. **(M)** *I. amethystoides*; **(N,O)** *I. serra*.

Nutlets of *Isodon* species are 0.9–2.1 mm long, 0.6–1.3 mm wide, and range from light yellow or yellowish brown to dark brown (Figure 3 and Supplementary Table 4). All species are characterized by ovate or rounded nutlets (length/width ≤ 1.8) having rounded apex (opposite to the areole area), except for *I. ternifolius*, which has trigonous-oblong nutlets (length/width ≥ 2) with acute apex. Six different surface types of nutlets can be recognized within *Isodon*, including psilate, reticulate, reticulate-papillate, striate, cellular, and glandular (and pubescent) types (Figure 4 and Supplementary Table 4). Both the psilate and reticulate types can be found in species of Clade I, and the reticulate-papillate type is confined to the two African species of Clade II. *Isodon ternifolius* (Clade III) has distinct nutlets with a striate surface, whereas most species of Clade IV possess nutlets with cellular surfaces. Nutlets with glandular or glandular and pubescent trichomes are only possessed by the remaining species of Clade IV.

## Discussion

### Phylogenetic relationship within *Isodon*

This examination of infrageneric relationships for *Isodon* was aimed at resolving the phylogenetic ambiguities found by prior phylogenetic studies. Our study has increased taxonomic sampling to include all four sections and ten series, and provided additional geographic sampling to accommodate the distribution of *Isodon*, especially from HM. Our results recover four monophyletic clades (Clades I–IV) of *Isodon* species similar to Yu et al. (2014). However, the combination of plastome-scale data and nrDNA sequences of *Isodon* with our extensive field investigation and morphological studies enables us to further discuss the relationships within each clade.

### Relationships within Clade I

All species recovered in Clade I are perennial herbs with reddish-brown glands all over the plants, the latter character was considered a useful synapomorphy of this clade by Zhong et al. (2010) and Yu et al. (2014), as opposed to the colorless glands in the remaining species of *Isodon* (Supplementary Figure 6). A third kind of glands was discovered in our recently published new species, *I. aurantiacus* Y.P. Chen and C.L. Xiang, from Tibet, China (Chen et al., 2017), which is embedded within Clade IV (Figure 1 and Supplementary Figure 5). The plants of this species are covered with orange glands (Supplementary Figure 6).

Two subclades can be further recognized within Clade I, whereas the placement of *I. scrophularioides* exhibits the largest discrepancy that it is recovered within different subclades in the plastid and nrDNA trees. The nutlet morphology seems to support the position of *scrophularioides* in the nrDNA tree. Our examination of the exine ornamentation on nutlets shows a close relationship between *I. oreophilus* and *I. phyllopodus* with *I. scrophularioides*, as their surfaces are reticulate, unlike the psilate surface sculpture in species (*I. atroruber* and *I. lophanthoides*) belonging to the other subclade of Clade I (Figure 4 and Supplementary Table 4). However, a more comprehensive sampling of species with reddish-brown glands is needed to verify the hypothesis.

Species of Clade I are most diverse in the Himalayas and tropical Asia. Based on our current understanding of the taxonomy of *Isodon*, a total of 20 species have been reported with reddish-brown glands (Li, 1988; Zhong et al., 2010; Chen et al., 2016b; Kumar et al., 2016; Ranjan et al., 2022). The continuous discovery of new species possessing this character during recent years possibly indicates an underestimation of the species diversity of Clade I.

### Synapomorphy of Clade II

The systematic placement of the two species of *Isodon* endemic to Africa, *I. ramosissimus* and *I. schimperi*, was first elucidated by Yu et al. (2014), using nrITS, three plastid markers, and a low-copy nuclear gene. Our results here are congruent with that of Yu et al. (2014) that the two species were recovered in a monophyletic clade either sister to the large clade formed by Clade III and Clade IV in the plastid tree (Figure 1) or Clade I in the nuclear tree (Supplementary Figure 5). Based on molecular phylogenetic analyses, chromosome evolution, and biogeographic study, Yu et al. (2014) inferred that the two African species were of hybrid origin between members of Clade I and Clades III–IV, both of which likely formed through allopolyploidy; an overland migration from Asia to Africa through Arabia in early to middle Miocene and the opening of the Red Sea in the middle Miocene probably resulted in the present disjunct distribution of *Isodon*.

Though the evolutionary history of Clade II has been elucidated by previous study, the synapomorphy of this clade was unclear. Both species are herbs with loose panicles, straight corolla tubes, and long exserted stamens, morphologically similar to *I. lophanthoides* and related species from Clade I but with colorless glands on the plants. Our morphological study of nutlets further support the distinctiveness of the two endemic species from Africa that they possess reticulate-papillate nutlet surfaces differing from all other species of *Isodon*, which can be regarded as a synapomorphy of this clade (Figure 4).

### Synapomorphy of Clade III

By including two accessions of *I. ternifolius* in the analysis, we provide additional evidence that supports *I. ternifolius* as a distinct lineage within *Isodon* (Zhong et al., 2010; Yu et al., 2014). *Isodon ternifolius*, which is widely distributed from tropical China to South and Southeast Asia (Li, 1988; Suddee et al., 2004), is reported by Zhong et al. (2010) to be distinguished by a 3–4-whorled leaf phyllotaxy. However, opposite phyllotaxy can be observed on some individuals of a whorled-leaf population of *I. ternifolius*, and sometimes even on an individual with whorled-leaves (Li, 1988; Suddee et al., 2004; Y.P. Chen, pers. obs.). Furthermore, whorled leaves can occasionally be found in other species of *Isodon* (Y.P. Chen, pers. obs.). Invariable transitions between phyllotaxy such as these indicate that the current diagnosis of *I. ternifolius* cannot be properly applied, and must be adjusted to a more stable synapomorphic character. Our examination of nutlets (Figures 3, 4) suggest that irrespective of phyllotaxy, *I. ternifolius* has trigonous-oblong nutlets with an acute apex, unlike the nutlets of other *Isodon* species, which are ovate and have an obtuse or rounded apex. Moreover, the striate nutlet ornamentation of *I. ternifolius* is also unique in the genus.

### Relationships within Clade IV

The majority (ca. 85%) of *Isodon* species in the phylogenetic trees are recovered in Clade IV. Despite that the relationships within this clade are much better resolved in the plastid tree (Figure 1), compared with that of nrDNA tree which primarily includes a large polytomy and scant substructures associated with short branch lengths (Supplementary Figure 5), strong cytoplasmic-nuclear discordance is also revealed (Figure 2). Moreover, some nodes within Clade IV are still poorly supported or show conflicts among the 12 concatenated trees, indicating that whole plastome sequence is not a panacea in resolving phylogenetic relationships within rapidly radiated genera.

As the most morphologically diverse group of *Isodon*, species of Clade IV are herbs or shrubs with a variety of leaves, inflorescences, and trichomes. It has been difficult to find out the synapomorphy for such a clade. Interestingly, herbal species are mostly distributed in eastern Asia and Japan, whereas the shrubby species, which account for a large proportion of Clade IV, are mainly accustomed to the dry valleys in HM. Two types of nutlet surface are restricted to Clade IV as revealed by our morphological study, with most species sharing the cellular nutlet ornamentation (Figure 4). The remaining species are characterized with glandular or glandular and pubescent nutlets; all these species [except for *I. tenuifolius* (W.W. Sm.) Kudô] are herbs from the forest area in eastern Asia and Japan.

### Causes of phylogenetic incongruences

The 12 concatenated plastome data sets resulted in largely congruent and better resolved topologies (Figure 1 and Supplementary Figure 3) than that of previous studies. However, it can be noted that the branch lengths within Clade IV are extremely short and some nodes receive weak support values or exhibit conflicts among these data sets, especially among different sequence types (coding vs. non-coding) (Supplementary Figure 3). Moreover, concordance analyses of single gene trees demonstrated that most of the plastid genes are uninformative for majority of nodes, but strongly supported conflict at some nodes is also recovered (Supplementary Figure 4). Systematic (e.g., modeling error, misalignment, saturation, and limited taxon sampling) and stochastic errors (e.g., limited phylogenetic signal/information) as well as biological factors (e.g., selection and possibility of genes with distinct evolutionary histories, incomplete lineage sorting) might potentially contribute to these observed discordances (Walker et al., 2019). Based on a comprehensive sampling and our efforts to prune regions with poor alignment and exclude loci with high levels of substitutional saturation, systematic errors fail to explain most of the conflicts as the resulting data sets recovered largely concordant infrageneric relationships (Supplementary Figure 3). Previous studies (Walker et al., 2019; Zhang et al., 2020) suggested that stochasticity could be a primary source of conflict among plastid loci. Phylogenetic relationships inferred from genes with limited information (usually with short length or slow-evolving) could be spurious and misleading. For a genus which has undergone recent rapid radiation, most single plastid regions harbor too few variable sites to be informative and thus unable to accurately infer relationships within *Isodon*. Although potential sources of conflict such as selection and heteroplasmy (i.e., distinct plastomes coexist within a single organism resulted from plastid recombination, transfer, and/or complex mutational dynamics) have been documented from many other taxa (Lee-Yaw et al., 2019; Ramsey and Mandel, 2019), they still warrant further investigation in *Isodon*.

From our analyses, it is clear that well-supported tree obtained from plastome data is in strong conflict with the tree inferred from nuclear data (Figure 2). Moreover, the infrageneric relationships recovered within the plastid tree are also incongruent with the current taxonomy (mainly based on morphology and ecology) and species distribution. Patterns of variation in nuclear markers often agree with morphologically defined species boundaries (e.g., Rautenberg et al., 2010; Schuster et al., 2018; Ogishima et al., 2019). However, the sister relationship of *I. interruptus* (C.Y. Wu and H.W. Li) H. Hara and *I. brachythyrsus* (C.Y. Wu and H.W. Li) H. Hara which receives high support (BS = 96%/PP = 1.00) in the nrDNA tree is in contrast with their significant differences in the morphology of inflorescence (spike-like panicles with bracts longer than cymes in *I. interruptus* vs. loose panicles with bracts shorter than cymes in *I. brachythyrsus*), calyx (tubular campanulate vs. broadly campanulate), and corolla (yellow with tube slightly saccate abaxially near base vs. white to pale lavender with tube calcarate abaxially near base), whereas *I. interruptus* shares all these features with *I. muliensis* (W.W. Sm.) Kudô (Chen and Xiang, 2019). The minor differences between *I. interruptus* and *I. muliensis* are that *I. interruptus* has shorter habits and petioles and smaller leaves. Despite the constraints of nrDNA sequences (e.g., multiple rDNA arrays, concerted evolution, pseudogenes, and secondary structure) (Álvarez and Wendel, 2003; Feliner and Rossello, 2007), the limited substructures recovered within Clade IV in the nuclear tree are mostly formed by species with similar morphology and from the same distribution area. In contrast, similar patterns of morphology and geographic distribution are hardly found in the plastid tree (Figure 1). For example, the two sister groups recovered in the nrDNA tree (Supplementary Figure 5), *I. gibbosus* (C.Y. Wu and H.W. Li) H. Hara and *I. lungshengensis* (C.Y. Wu and H.W. Li) H. Hara, *I. xerophilus* (C.Y. Wu and H.W. Li) H. Hara and *I. enanderianus* (Hand.-Mazz.) H.W. Li, can also be supported by their shared morphology, habitat, and distribution. Plants of *I. gibbosus* and *I. lungshengensis* are perennial herbs usually grow at moist streamside in the bordering area of Guangxi, Hunan, and Guizhou Provinces, China, while *I. enanderianus* and *I. xerophilus* are shrubs restricted to the dry valleys in southern Yunnan Province (Wu and Li, 1977; Li and Hedge, 1994). However, in the plastid tree, *I. gibbosus* falls sister to *I. enanderianus*, both of which are distantly related to *I. lungshengensis* and *I. xerophilus* (Figure 1).

Discordances between nuclear tree/morphological taxonomy and plastid tree are much often attributed to interspecific hybridization, which could be the gene flow in the nuclear genome or plastid capture. The discordant placement of the two African species (Clade II) and *I. scrophularioides* likely indicates a hybrid origin, as has been shown in Yu et al. (2014). Except for these species, several natural hybrids have been described from Japan (Murata and Yamazaki, 1993); our recent field investigations have also detected some putative hybrids in sympatric populations of *Isodon* that will require further study (Chen, 2017; Y.P. Chen, pers. obs.). Although genetic exchange within the genus seems to be frequent, both chromosome incompatibilities and/or backcrossing with one parent could erased most signals of gene flow in the nuclear genome, but these signals may have been retained in the plastid genome by chance or by selection. Moreover, in seed plants with maternally inherited organelles, interspecific exchange of plastid loci occurs more frequently than that of nuclear genes (Rieseberg and Soltis, 1991). Therefore, the majority of topological conflicts between the plastid and nuclear trees possibly is a consequence of plastid capture, i.e., the plastid genome of one species is replaced by an alien one through intrageneric hybridization and introgression as well as recurrent backcrossing, while the nuclear genome remains largely unchanged (Rieseberg and Soltis, 1991; Tsitrone et al., 2003; Chan and Levin, 2005; Stegemann et al., 2012). Molecular phylogenetic studies of Japanese *Isodon* also revealed that most populations belonging to the same species are rarely monophyletic in the plastid tree, but group together in the nuclear tree, indicating that plastid capture may have frequently occurred between *Isodon* species from Japan (Maki et al., 2010; Ogishima et al., 2019). Previous studies frequently recovered geographic patterns of large-scale structuring in plastid haplotypes (e.g., Rautenberg et al., 2010; Pham et al., 2017; Schuster et al., 2018). However, this geographic congruence with infrageneric relationships in the plastid tree is neither supported by our result nor by the analyses of Japanese *Isodon* (Maki et al., 2010; Ogishima et al., 2019). Most species forming monophyletic clades/sister groups in the plastid tree do not show sympatric distribution or geographic proximity (Figure 1), suggesting the plastid capture might be a consequence of ancient (rather than ongoing) interspecific hybridization within shared areas in the past.

Taken as a whole, our results suggest that stochasticity stemming from recent rapid radiation and limited phylogenetic signal, widespread hybridization and plastid capture, independently or in concert, may contribute to a complex evolutionary history of *Isodon*. Moreover, our results demonstrate that plastome data does not accurately reflect species relationships and is insufficient to resolve shallow level relationships within a rapidly radiated genus like *Isodon*. To achieve for a better resolved phylogeny of the genus, significant larger data sets derived from nuclear genome with next-generation sequencing technology might be helpful, such as targeted enrichment (Gardner et al., 2020; Reichelt et al., 2021; Giaretta et al., 2022), deep genome skimming (Liu et al., 2021), and RNA-seq (Nevado et al., 2016; Jin et al., 2021; Kong et al., 2022; Xia et al., 2022), which are compatible with more comprehensive tools for disambiguating genetic lineages and causes of gene tree conflicts.

## Conclusion

*Isodon*, as revealed by plastid and nuclear phylogenetic data, is comprised of four clades, the fourth (Clade IV) of which consists of the largest portion of *Isodon* (85% of species). The tree topology at deep nodes largely corresponds with previous molecular phylogenetic studies of *Isodon*, whereas we further reveal that Clade II which consisting of the two African endemic species differs from the remaining *Isodon* species by the reticulate-papillate nutlet surfaces. Clade III is composed of individuals of *I. ternifolius* with both whorled and opposite phyllotaxy, while our morphological examination demonstrates that nutlets with acute and conspicuously trigonous apex is exclusive to all individuals of *I. ternifolius*. Phylogenetic relationships within Clade IV are much better resolved in the plastid tree than that in previous studies, but highly incongruent with the nrDNA tree and morphology and distribution. Species of Clade IV have cellular nutlet surface type and the distinct nutlets with trichomes, the latter type is restricted to species mostly from eastern Asia and Japan. Our results also suggest that multiple processes might have been involved in the evolutionary history of *Isodon* and plastome sequences fail to further resolve the shallow-level relationships within the genus. Large-scale molecular markers from the nuclear genome might contribute to a better understanding of the infrageneric relationships of the rapidly radiated *Isodon*.

## Data availability statement

The original contributions presented in this study are publicly available. The raw sequence data were deposited in the NCBI BioProject database under the accession number: PRJNA863331, and the sequence alignments and resulting tree files were deposited in figshare at https://doi.org/10.6084/m9.figshare.20170727.

## Author contributions

Y-PC, L-MG, and C-LX conceived this study. Y-PC and FZ analyzed the data. Y-PC drafted the manuscript with contributions from other authors. All authors collected the materials, read, and approved the final manuscript.

## Footnotes

1
http://www.phylo.org/

2
https://github.com/mossmatters/MJPythonNotebooks

## Supplementary material

Supplementary Figure 1 Locus-specific saturation indices for 207 loci shown as density plots (distribution). **(A)** *R*^2^ of the linear regression between patristic and uncorrected pairwise distances. **(B)** Slopes of the linear regression between patristic and uncorrected pairwise distances. Dashed line indicates starting shoulder value. Red regions on the left-hand side contain loci that might be saturated.

Supplementary Figure 2 Gene map of the complete plastomes of *Isodon* species. Genes inside and outside of the circle are transcribed in the clockwise and counterclockwise directions, respectively. Genes belonging to different functional categories are color-coded. LSC, large single copy; SSC, small single copy; IR, inverted repeat.

Supplementary Figure 3 Cladogram of the maximum-likelihood tree estimated from the CR + NCR-GB data set used as a reference to evaluate conflict and concordance among the trees estimate from the remaining 11 data sets. Pie charts depict conflict amongst the input trees, with the blue, green, red, and gray slices representing the proportion of input bipartitions concordant, conflicting (supporting a single main alternative topology), conflicting (supporting various alternative topologies), and uninformative (< 70% BS) at each node in the CR + NCR-GB tree, respectively. The numbers above and below each branch are the number of bipartitions concordant and conflicting with that particular node, respectively.

Supplementary Figure 4 Summary of gene tree conflict against the reference CR + NCR-GB tree. Pie charts depict conflict amongst the input locus trees, with the blue, green, red, and gray slices representing the proportion of the 207 trees concordant, conflicting (supporting a single main alternative topology), conflicting (supporting various alternative topologies), and uninformative (< 70% BS) at each node in the PCN tree, respectively. The numbers above and below each branch are the number of bipartitions concordant and conflicting with that particular node, respectively.

Supplementary Figure 5 Phylogram of the maximum-likelihood tree of *Isodon* inferred from the nrDNA data set. Support values ≥ 50% BS or 0.50 PP are displayed above the branches (“*” indicates a support value = 100% BS or 1.00 PP, “–” indicates a support value < 0.50 PP).

Supplementary Figure 6 Glands with different colors on leaves and calyces of *Isodon* species. **(A,B)** reddish-brown, *I. lophanthoides* var. *lophanthoides*; **(C,D)** colorless, I. serra; **(E,F)** orange, *I. aurantiacus*.
