# Supplementary figures and images for: Plastome sequences fail to resolve shallow level relationships within the rapidly radiated genus Isodon (Lamiaceae)
Source: Front Plant Sci. 2022 Sep 8;13:985488. doi: 10.3389/fpls.2022.985488 (PMC9493350; doi:10.3389/fpls.2022.985488)

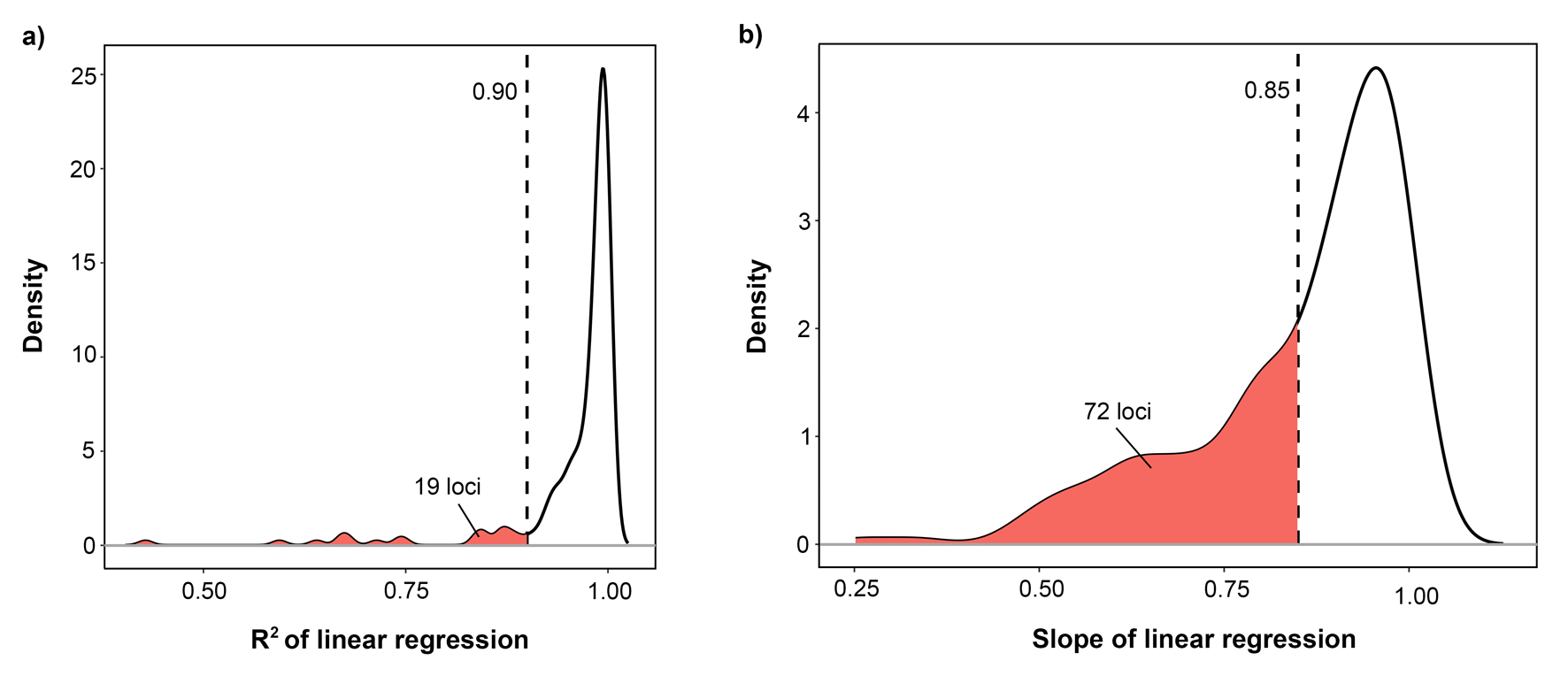

Supplement: Supplementary Figure 1 — Locus-specific saturation indices for 207 loci shown as density plots (distribution). (A) R2 of the linear regression between patristic and uncorrected pairwise distances. (B) Slopes of the linear regression between patristic and uncorrected pairwise distances. Dashed line indicates starting shoulder value. Red regions on the left-hand side contain loci that might be saturated. [file Image_1.JPEG]

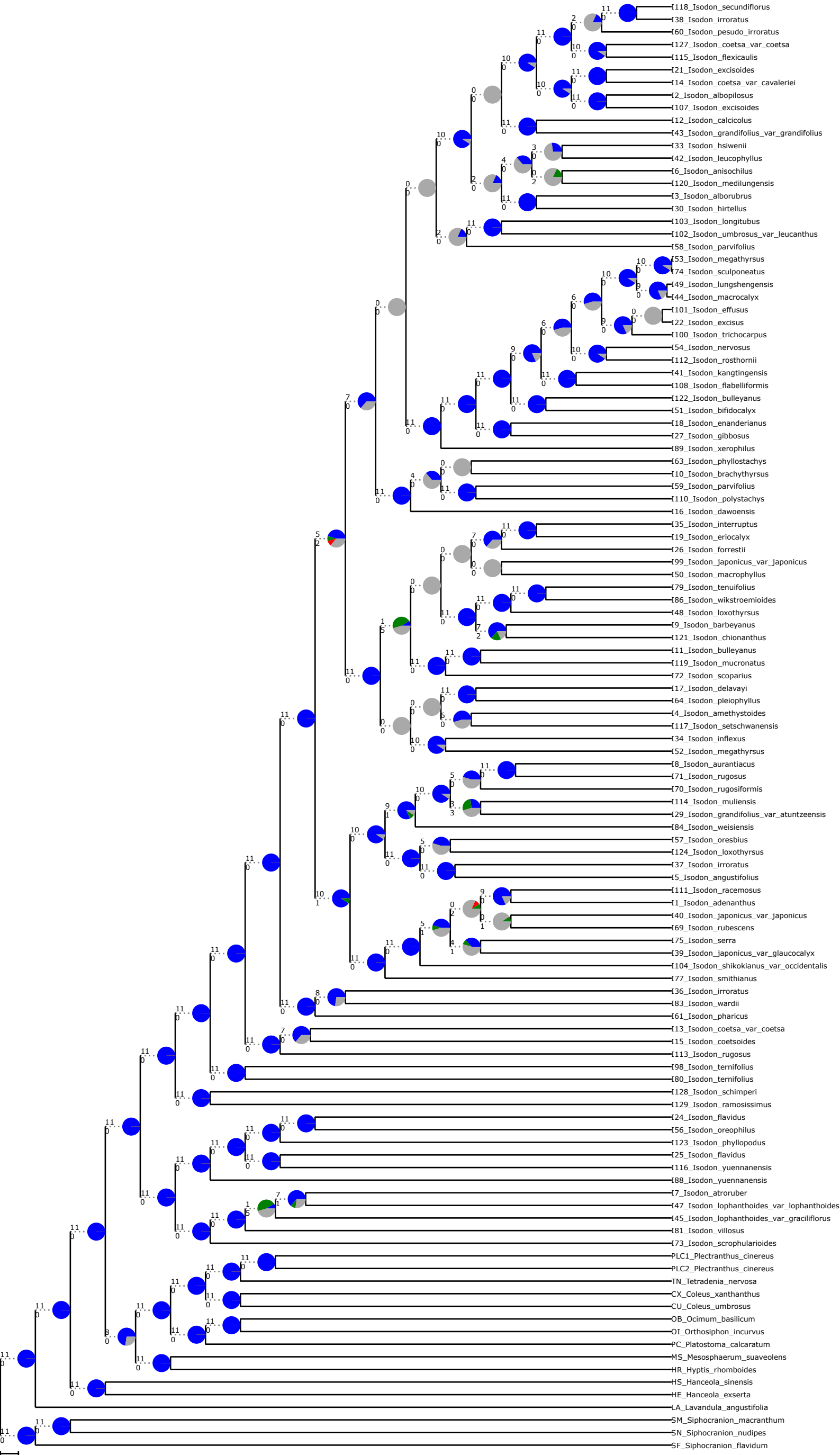

Supplement: Supplementary Figure 3 — Cladogram of the maximum-likelihood tree estimated from the CR + NCR-GB data set used as a reference to evaluate conflict and concordance among the trees estimate from the remaining 11 data sets. Pie charts depict conflict amongst the input trees, with the blue, green, red, and gray slices representing the proportion of input bipartitions concordant, conflicting (supporting a single main alternative topology), conflicting (supporting various alternative topologies), and uninformative (< 70% BS) at each node in the CR + NCR-GB tree, respectively. The numbers above and below each branch are the number of bipartitions concordant and conflicting with that particular node, respectively. [file Image_3.PDF]

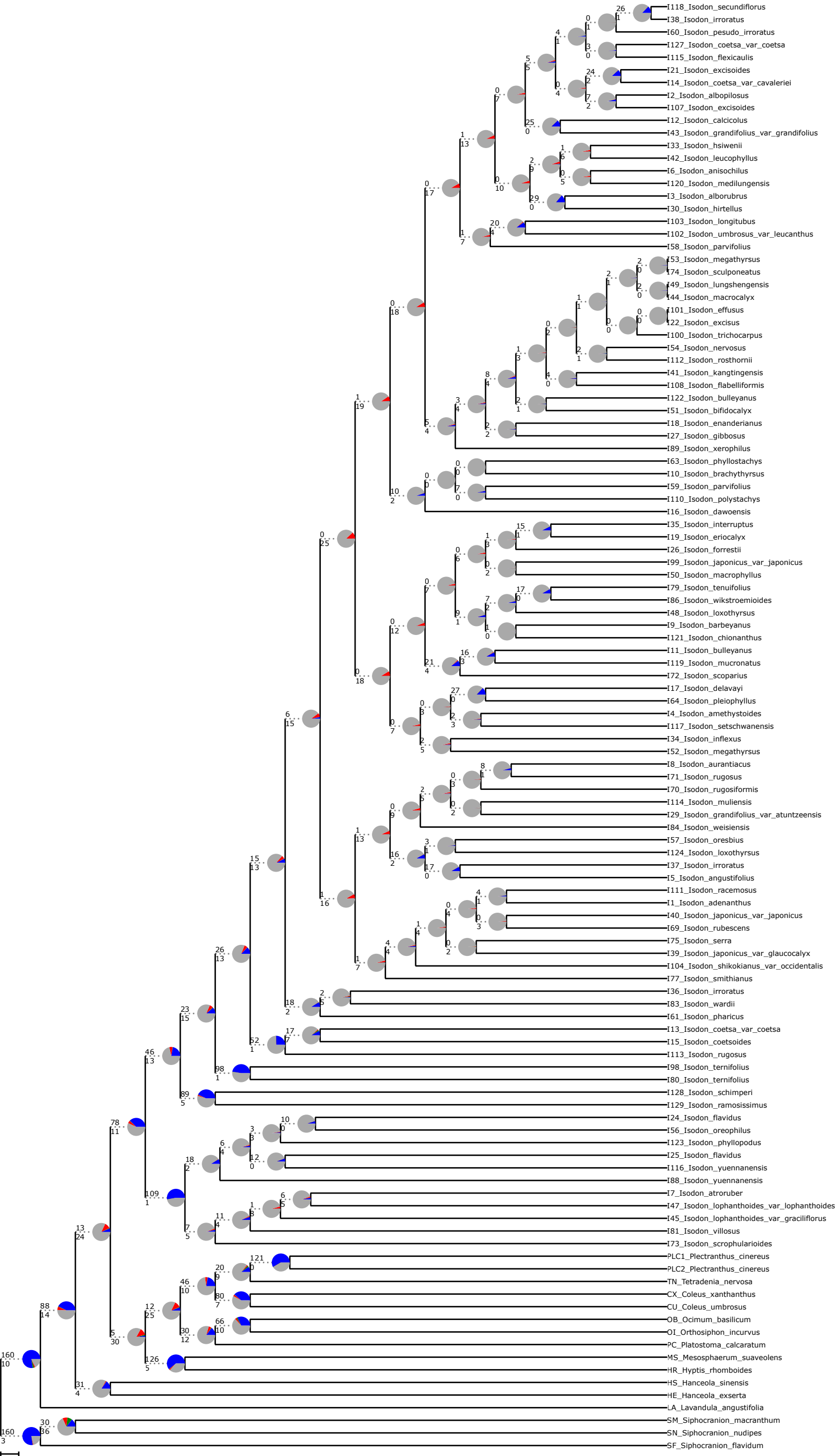

Supplement: Supplementary Figure 4 — Summary of gene tree conflict against the reference CR + NCR-GB tree. Pie charts depict conflict amongst the input locus trees, with the blue, green, red, and gray slices representing the proportion of the 207 trees concordant, conflicting (supporting a single main alternative topology), conflicting (supporting various alternative topologies), and uninformative (< 70% BS) at each node in the PCN tree, respectively. The numbers above and below each branch are the number of bipartitions concordant and conflicting with that particular node, respectively. [file Image_4.PDF]

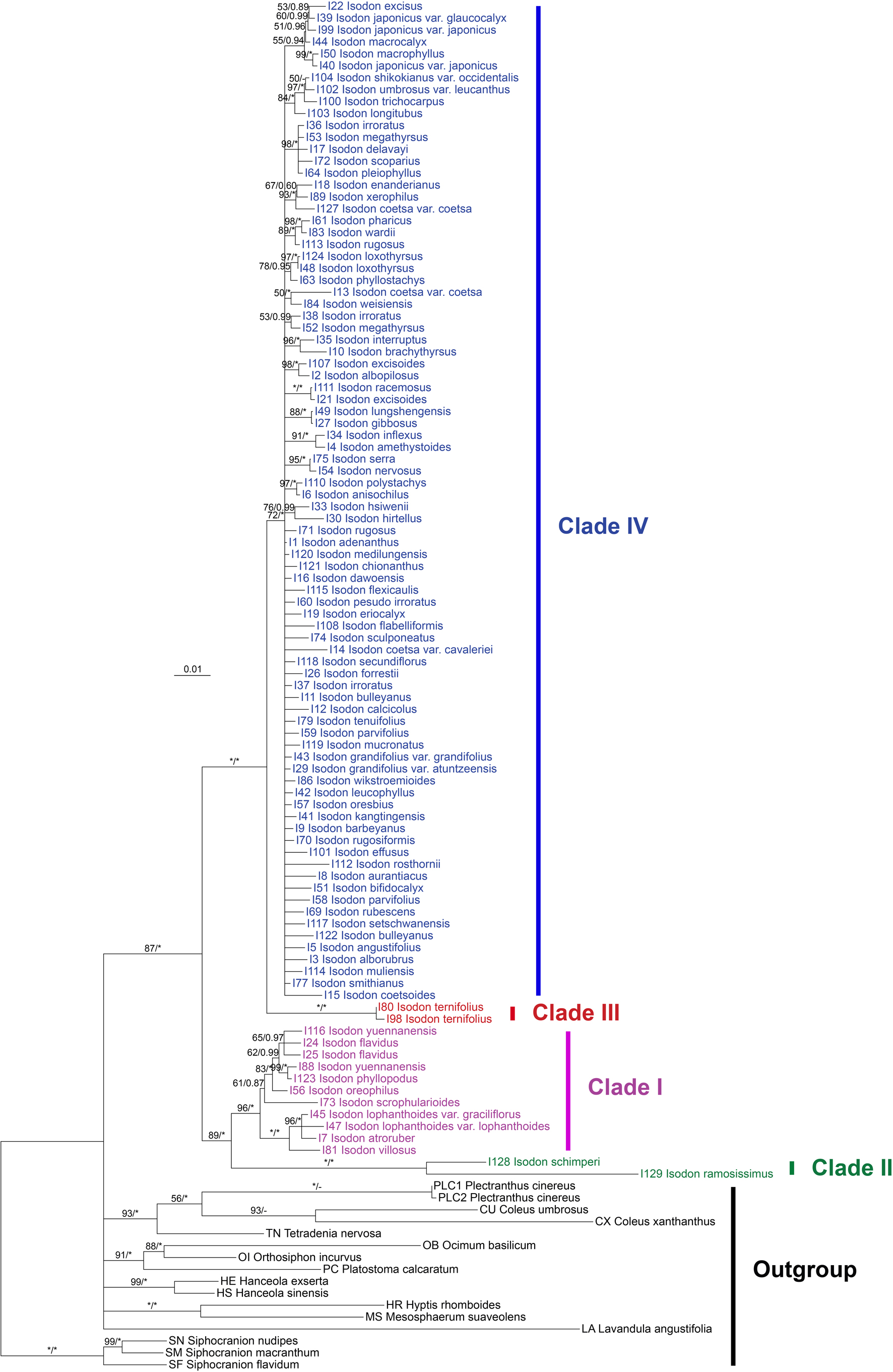

Supplement: Supplementary Figure 5 — Phylogram of the maximum-likelihood tree of Isodon inferred from the nrDNA data set. Support values ≥ 50% BS or 0.50 PP are displayed above the branches (“*” indicates a support value = 100% BS or 1.00 PP, “–” indicates a support value < 0.50 PP). [file Image_5.JPEG]

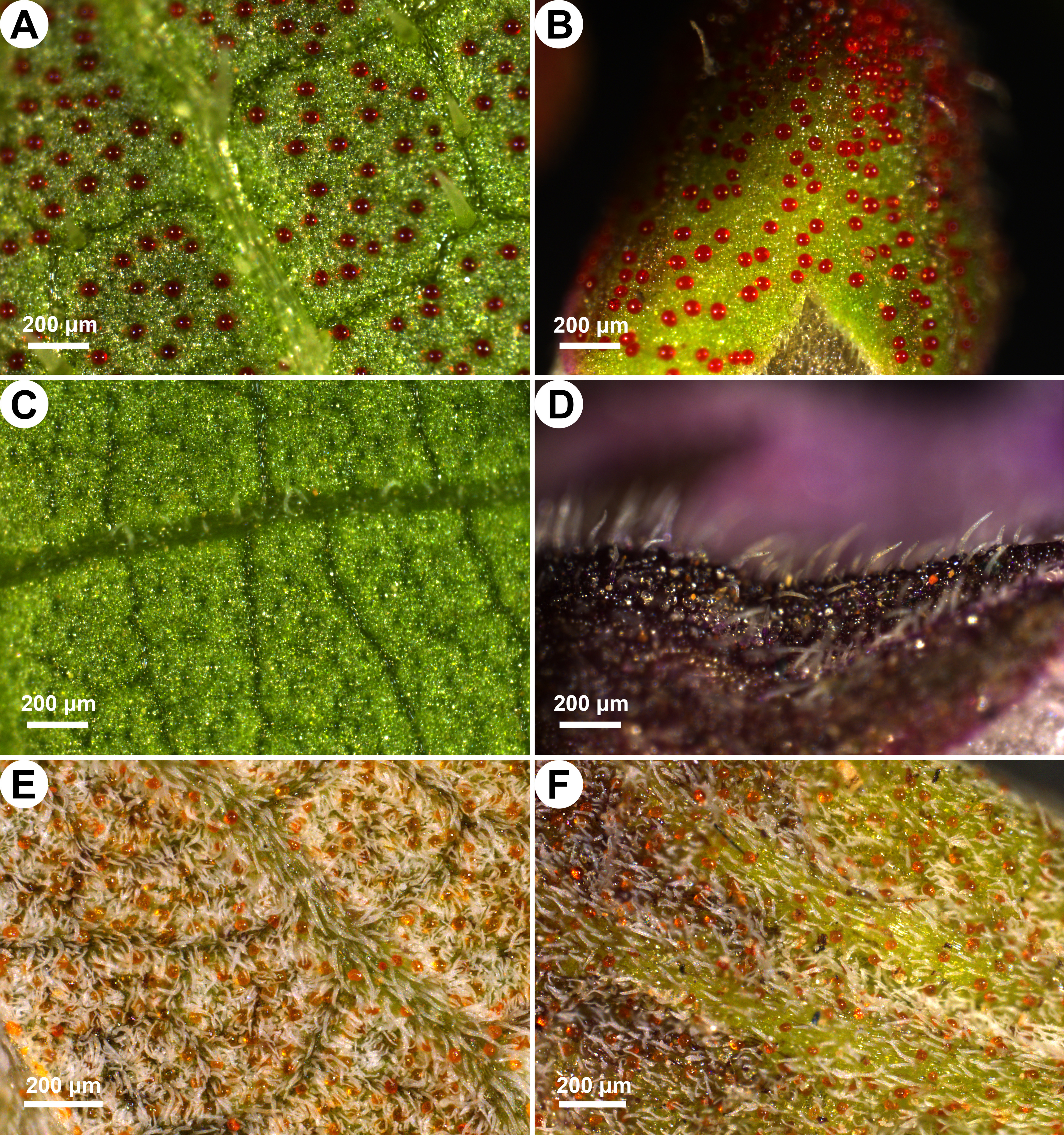

Supplement: Supplementary Figure 6 — Glands with different colors on leaves and calyces of Isodon species. (A,B) reddish-brown, I. lophanthoides var. lophanthoides; (C,D) colorless, I. serra; (E,F) orange, I. aurantiacus. [file Image_6.JPEG]
