# Supplementary material for: Plastome sequences fail to resolve shallow level relationships within the rapidly radiated genus Isodon (Lamiaceae)
Source: Front Plant Sci. 2022 Sep 8;13:985488. doi: 10.3389/fpls.2022.985488 (PMC9493350; doi:10.3389/fpls.2022.985488)
Supplement: Supplementary Figure 2 — Gene map of the complete plastomes of Isodon species. Genes inside and outside of the circle are transcribed in the clockwise and counterclockwise directions, respectively. Genes belonging to different functional categories are color-coded. LSC, large single copy; SSC, small single copy; IR, inverted repeat. [file Image_2.PDF]

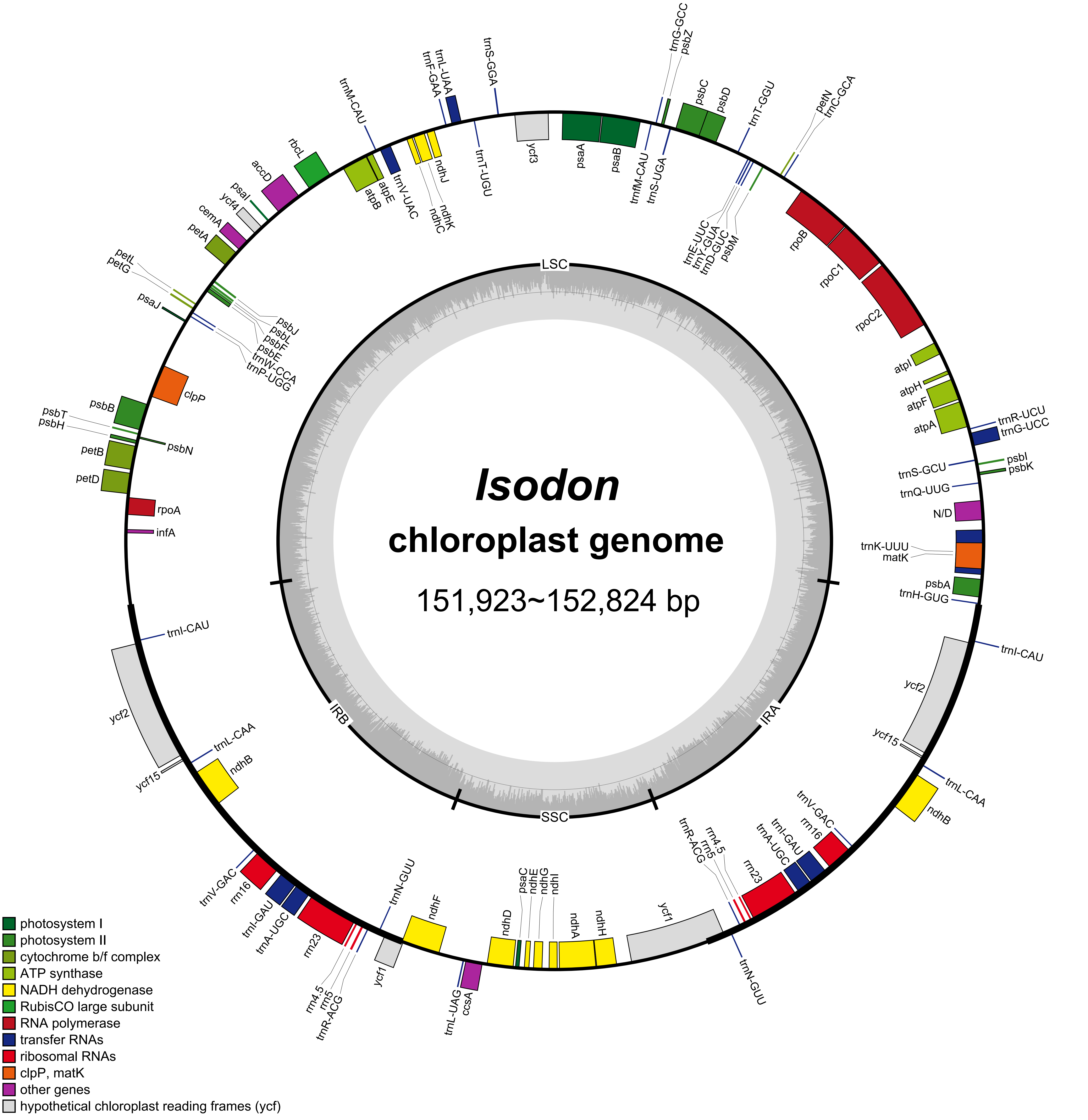

Isodon
chloroplast genome
151,923~152,824 bp
LSC
IRA
SSC
IRB
trnG-GCC
psbZ
psbC
psbD
trnT-GGU
petN
trnC-GCA
trnS-GGA
trnL-UAA
trnF-GAA
trnM-CAU
rbcL
accD
psaI
ycf4
cemA
petA
petL
petG
psaJ
psbJ
psbL
psbF
psbE
trnW-CCA
trnP-UGG
clpP
psbB
psbT
psbH
psbN
petB
petD
rpoA
infA
ycf3
psaA
psaB
trnfM-CAU
trnS-UGA
trnE-UUC
trnY-GUA
trnD-GUC
psbM
trnT-UGU
ndhJ
ndhK
ndhC
trnV-UAC
atpE
atpB
rpoB
rpoC1
rpoC2
atpI
atpH
atpF
atpA
trnR-UCU
trnG-UCC
psbI
psbK
trnS-GCU
trnQ-UUG
N/D
trnK-UUU
matK
psbA
trnH-GUG
trnI-CAU
ycf2
ycf15
trnL-CAA
ndhB
trnV-GAC
rrn16
trnI-GAU
trnA-UGC
rrn23
rrn4.5
rrn5
trnR-ACG
trnN-GUU
ycf1
ndhF
trnL-UAG
ccsA
ndhD
psaC
ndhE
ndhG
ndhI
ndhA
ndhH
photosystem I
photosystem II
cytochrome b/f complex
ATP synthase
NADH dehydrogenase
RubisCO large subunit
RNA polymerase
transfer RNAs
ribosomal RNAs
clpP, matK
other genes
hypothetical chloroplast reading frames (ycf)
