# Supplementary material for: Plastome sequences fail to resolve shallow level relationships within the rapidly radiated genus Isodon (Lamiaceae)
Source: Front Plant Sci. 2022 Sep 8;13:985488. doi: 10.3389/fpls.2022.985488 (PMC9493350; doi:10.3389/fpls.2022.985488)
Supplement: Supplementary file 7 [file Table_1.DOCX]

**TABLE S1.** Sequence information for all samples used in present study. “-” indicates missing data. Sequences downloaded from GenBank are marked in bold. Herbarium abbreviations are listed after the vouchers.

| Taxon | Voucher | Country | Plastome | ETS | ITS |
| --- | --- | --- | --- | --- | --- |
| I1_Isodon_adenanthus | Y.P. Chen et al. EM339 (KUN) | Shiping, Yunnan, China | **ON858832** | - | **ON890682** |
| I2_Isodon_albopilosus | W. Fang et al. FW11202 (KUN) | Diebu, Gansu, China | **ON858833** | MG232738 | **ON890717** |
| I3_Isodon_alborubrus | Y.P. Chen et al. EM445 (KUN) | Yuanmou, Yunnan, China | **ON858834** | **ON854763** | **ON890725** |
| I4_Isodon_amethystoides | Y.P. Chen et al. EM039 (KUN) | Lin'an, Zhejiang, China | MT473767 | **ON854770** | **ON890734** |
| I5_Isodon_angustifolius | C.L. Xiang et al. XCL1342 (KUN) | Heqing, Yunnan, China | **ON858835** | **ON854778** | **ON890744** |
| I6_Isodon_anisochilus | E.D. Liu et al. 2905 (KUN) | Muli, Sichuan, China | **ON858836** | MG232695 | **ON890754** |
| I7_Isodon_atroruber | C.L. Xiang et al. XCL1510 (KUN) | Cona, Tibet, China | **ON858837** | - | **ON890760** |
| I8_Isodon_aurantiacus | C.L. Xiang et al. XCL1488 (KUN) | Milin, Tibet, China | **ON858838** | - | **ON890769** |
| I9_Isodon_barbeyanus | W. Fang et al. FW11244 (KUN) | Danba, Sichuan, China | **ON858839** | MG232696 | **ON890777** |
| I10_Isodon_brachythyrsus | C.L. Xiang et al. XCL1642 (KUN) | Muli, Sichuan, China | **ON858840** | MG232682 | **ON890683** |
| I11_Isodon_bulleyanus | Y.P. Chen et al. EM222 (KUN) | Dali, Yunnan, China | **ON858841** | **ON854740** | **ON890691** |
| I12_Isodon_calcicolus | C.L. Xiang et al. XCL1350 (KUN) | Eryuan, Yunnan, China | **ON858842** | **ON854746** | **ON890702** |
| I13_Isodon_coetsa_var_coetsa | C.L. Xiang et al. XCL1521 (KUN) | Cona, Tibet, China | **ON858843** | **ON854755** | **ON890710** |
| I14_Isodon_coetsa_var_cavaleriei | Y.P. Chen et al. EM241 (KUN) | Qiaojia, Yunnan, China | **ON858844** | MG232666 | **ON890711** |
| I15_Isodon_coetsoides | Y.P. Chen et al. EM161 (KUN) | Longling, Yunnan, China | **ON858845** | **ON854756** | **ON890712** |
| I16_Isodon_dawoensis | E.D. Liu et al. 3226 (KUN) | Luhuo, Sichuan, China | **ON858846** | MG232710 | **ON890713** |
| I17_Isodon_delavayi | Y.P. Chen et al. EM457 (KUN) | Eryuan, Yunnan, China | **ON858847** | **ON854757** | **ON890714** |
| I18_Isodon_enanderianus | Y.P. Chen et al. EM344 (KUN) | Xinping, Yunnan, China | **ON858848** | **ON854758** | **ON890715** |
| I19_Isodon_eriocalyx | Y.P. Chen et al. EM317 (KUN) | Binchuan, Yunnan, China | **ON858849** | **ON854759** | **ON890716** |
| I21_Isodon_excisoides | Y.P. Chen et al. EM072 (KUN) | Shennongjia, Hubei, China | **ON858850** | **ON854760** | **ON890718** |
| I22_Isodon_excisus | Y.P. Chen et al. EM215 (KUN) | Changbaishan, Jilin, China | **ON858851** | MG232660 | **ON890719** |
| I24_Isodon_flavidus | Y.P. Chen et al. EM338 (KUN) | Shiping, Yunnan, China | **ON858852** | MG232686 | **ON890720** |
| I25_Isodon_flavidus | Z.H. Wang et al. WZH113 (KUN) | Jingdong, Yunnan, China | **ON858853** | **ON854761** | **ON890721** |
| I26_Isodon_forrestii | E.D. Liu et al. 2897 (KUN) | Muli, Sichuan, China | **ON858854** | MG232716 | **ON890722** |
| I27_Isodon_gibbosus | Y.P. Chen et al. EM431 (KUN) | Jiangkou, Guizhou, China | **ON858855** | MH557885 | **ON890723** |
| I29_Isodon_grandifolius_var_atuntzeensis | C.L. Xiang et al. XCL1407 (KUN) | Markam, Tibet, China | **ON858856** | **ON854762** | **ON890724** |
| I30_Isodon_hirtellus | Y.P. Chen et al. EM423 (KUN) | Luquan, Yunnan, China | **ON858857** | **ON854764** | **ON890726** |
| I33_Isodon_hsiwenii | C.L. Xiang et al. XCL1666 (KUN) | Ludian, Yunnan, China | **ON858858** | **ON854765** | **ON890727** |
| I34_Isodon_inflexus | Y.P. Chen et al. EM364 (KUN) | Yantai, Shandong, China | **ON858859** | **ON854766** | **ON890728** |
| I35_Isodon_interruptus | Y.P. Chen et al. EM130 (KUN) | Kunming, Yunnan, China | **ON858860** | MG232687 | **ON890729** |
| I36_Isodon_irroratus | Y.P. Chen et al. EM208 (KUN) | Lijiang, Yunnan, China | **ON858861** | MG232685 | **ON890730** |
| I37_Isodon_irroratus | C.L. Xiang et al. XCL1352 (KUN) | Eryuan, Yunnan, China | **ON858862** | **ON854767** | **ON890731** |
| I38_Isodon_irroratus | Y.P. Chen et al. EM329 (KUN) | Binchuan, Yunnan, China | **ON858863** | **ON854768** | **ON890732** |
| I39_Isodon_japonicus_var_glaucocalyx | Y.P. Chen et al. EM220 (KUN) | Changchong, Jilin, China | **ON858864** | **ON854769** | **ON890733** |
| I40_Isodon_japonicus_var_japonicus | E.D. Liu et al. 3097 (KUN) | Wenxian, Gansu, China | **ON858865** | MG232688 | **ON890735** |
| I41_Isodon_kangtingensis | C.L. Xiang et al. XCL1635 (KUN) | Muli, Sichuan, China | **ON858866** | **ON854771** | **ON890736** |
| I42_Isodon_leucophyllus | E.D. Liu et al. 3204 (KUN) | Jinchuan, Sichuan, China | **ON858867** | **ON854772** | **ON890737** |
| I43_Isodon_grandifolius_var_grandifolius | Y.P. Chen et al. EM265 (KUN) | Shangri-La, Yunnan, China | **ON858868** | **ON854773** | **ON890738** |
| I44_Isodon_macrocalyx | Y.P. Chen EM224 (KUN) | Hezhou, Guangxi, China | **ON858869** | **ON854774** | **ON890739** |
| I45_Isodon_lophanthoides_var_graciliflorus | Y.P. Chen et al. EM147 (KUN) | Yongsheng, Yunnan, China | **ON858870** | **ON854775** | **ON890740** |
| I47_Isodon_lophanthoides_var_lophanthoides | Y.P. Chen EM367 (KUN) | Kuming, Yunnan, China | MT473768 | **ON854776** | **ON890741** |
| I48_Isodon_loxothyrsus | C.L. Xiang et al. XCL1654 (KUN) | Shangri-La, Yunnan, China | **ON858871** | **ON854777** | **ON890742** |
| I49_Isodon_lungshengensis | Y.P. Chen et al. EM084 (KUN) | Lingui, Guangxi, China | **ON858872** | - | **ON890743** |
| I50_Isodon_macrophyllus | Y.P. Chen et al. EM034 (KUN) | Nanjing, Jiangsu, China | **ON858873** | MG232651 | **ON890745** |
| I51_Isodon_bifidocalyx | Y.P. Chen et al. EM040 (KUN) | Lin'an, Zhejiang, China | **ON858874** | MG232653 | **ON890746** |
| I52_Isodon_megathyrsus | H.J. Dong et al. D641 (KUN) | Eryuan, Yunnan, China | **ON858875** | MG232706 | **ON890747** |
| I53_Isodon_megathyrsus | C.L. Xiang et al. XCL1212 (KUN) | Muli, Sichuan, China | **ON858876** | **ON854779** | **ON890748** |
| I54_Isodon_nervosus | Y.P. Chen et al. EM444 (KUN) | Jiangkou, Guizhou, China | **ON858877** | MT614334 | **ON890749** |
| I56_Isodon_oreophilus | Y.P. Chen et al. EM354 (KUN) | Dali, Yunnan, China | **ON858878** | **ON854780** | **ON890750** |
| I57_Isodon_oresbius | H.J. Dong et al. D565 (KUN) | Shangri-La, Yunnan, China | **ON858879** | MG232732 | **ON890751** |
| I58_Isodon_parvifolius | C.L. Xiang et al. XCL1105 (KUN) | Tianshui, Gansu, China | **ON858880** | **ON854781** | **ON890752** |
| I59_Isodon_parvifolius | C.L. Xiang et al. XCL1609 (KUN) | Markam, Tibet, China | **ON858881** | **ON854782** | **ON890753** |
| I60_Isodon_pesudoirroratus | C.L. Xiang et al. XCL1625 (KUN) | Daocheng, Sichuan, China | **ON858882** | MG232681 | **ON890755** |
| I61_Isodon_pharicus | C.L. Xiang et al. XCL1548 (KUN) | Lhasa, Tibet, China | **ON858883** | **ON854783** | **ON890756** |
| I63_Isodon_phyllostachys | C.L. Xiang et al. XCL1335 (KUN) | Dali, Yunnan, China | **ON858884** | MG232690 | **ON890757** |
| I64_Isodon_pleiophyllus | Y.P. Chen et al. EM206 (KUN) | Lijiang, Yunnan, China | **ON858885** | **ON854784** | **ON890758** |
| I69_Isodon_rubescens | Y.P. Chen et al. EM073 (KUN) | Shennongjia, Hubei, China | **ON858886** | **ON854785** | **ON890759** |
| I70_Isodon_rugosiformis | Y.P. Chen et al. EM259 (KUN) | Deqin, Yunnan, China | **ON858887** | MG232684 | **ON890761** |
| I71_Isodon_rugosus | C.L. Xiang et al. XCL1492 (KUN) | Gyaca, Tibet, China | **ON858888** | **ON854786** | **ON890762** |
| I72_Isodon_scoparius | C.L. Xiang et al. XCL1362 (KUN) | Shangri-La, Yunnan, China | **ON858889** | **ON854787** | **ON890763** |
| I73_Isodon_scrophularioides | C.L. Xiang et al. XCL1526 (KUN) | Cona, Tibet, China | **ON858890** | **ON854788** | **ON890764** |
| I74_Isodon_sculponeatus | C.L. Xiang et al. XCL1346 (KUN) | Heqing, Yunnan, China | **ON858891** | **ON854789** | **ON890765** |
| I75_Isodon_serra | Y.P. Chen et al. EM037 (KUN) | Nanjing, Jiangsu, China | **ON858892** | MG232652 | **ON890766** |
| I77_Isodon_smithianus | E.D. Liu et al. 3186 (KUN) | Barkam, Sichuan, China | **ON858893** | MG232721 | **ON890767** |
| I79_Isodon_tenuifolius | C.L. Xiang et al. XCL1378 (KUN) | Deqin, Yunnan, China | **ON858894** | MG232679 | **ON890768** |
| I80_Isodon_ternifolius | C.L. Xiang et al. XCL1738 (KUN) | Malipo, Yunnan, China | **ON858895** | **ON854790** | **ON890770** |
| I81_Isodon_villosus | Y.P. Chen et al. EM227 (KUN) | Hezhou, Guangxi, China | **ON858896** | MG232662 | **ON890771** |
| I83_Isodon_wardii | C.L. Xiang et al. XCL1485 (KUN) | Milin, Tibet, China | **ON858897** | **ON854791** | **ON890772** |
| I84_Isodon_weisiensis | E.D. Liu et al. 3343 (KUN) | Deqin, Yunnan, China | **ON858898** | MG232713 | **ON890773** |
| I86_Isodon_wikstroemioides | Y.P. Chen et al. EM252 (KUN) | Shangri-La, Yunnan, China | **ON858899** | **ON854792** | **ON890774** |
| I88_Isodon_yuennanensis | C.L. Xiang et al. XCL1365 (KUN) | Shangri-La, Yunnan, China | **ON858900** | **ON854793** | **ON890775** |
| I89_Isodon_xerophilus | Y.P. Chen et al. EM346 (KUN) | Honghe, Yunnan, China | **ON858901** | **ON854794** | **ON890776** |
| I98_Isodon_ternifolius | C.L. Xiang et al. XCL1705 (KUN) | Wangmo, Guizhou, China | **ON858902** | MH557889 | **ON890778** |
| I99_Isodon_japonicus_var_japonicus | Y.P. Chen et al. EM370 (KUN) | Koishikawa Botanical Garden (cultivated) | **ON858903** | MH557895 | **ON890779** |
| I100_Isodon_trichocarpus | Y.P. Chen et al. EM371 (KUN) | Koishikawa Botanical Garden (cultivated) | **ON858904** | MH557894 | **ON890684** |
| I101_Isodon_effusus | Y.P. Chen et al. EM380 (KUN) | Okutama, Tokyo, Japan | **ON858905** | MH557896 | **ON890685** |
| I102_Isodon_umbrosus_var_leucanthus | Y.P. Chen et al. EM381 (KUN) | Okutama, Tokyo, Japan | **ON858906** | **ON854736** | **ON890686** |
| I103_Isodon_longitubus | Y.P. Chen et al. EM401 (KUN) | Kyoto Botanical Garden (cultivated) | **ON858907** | MH557898 | **ON890687** |
| I104_Isodon_shikokianus_var_occidentalis | Y.P. Chen et al. EM402 (KUN) | Kyoto Botanical Garden (cultivated) | **ON858908** | **ON854737** | **ON890688** |
| I107_Isodon_excisoides | Y.P. Chen et al. EM626 (KUN) | Emeishan, Sichuan, China | **ON858909** | **ON854738** | **ON890689** |
| I108_Isodon_flabelliformis | Y.P. Chen et al. EM716 (KUN) | Binchuan, Yunnan, China | **ON858910** | **ON854739** | **ON890690** |
| I110_Isodon_polystachys | Y.P. Chen et al. EM595 (KUN) | Kunming, Yunnan, China | **ON858911** | **ON854741** | **ON890692** |
| I111_Isodon_racemosus | Y.P. Chen et al. EM603 (KUN) | Yichang, Hubei, China | **ON858912** | **ON854742** | **ON890693** |
| I112_Isodon_rosthornii | Y.P. Chen et al. EM624 (KUN) | Emeishan, Sichuan, China | **ON858913** | **ON854743** | **ON890694** |
| I113_Isodon_rugosus | C. Liu et al. 18CS17466 (KUN) | Jirong, Tibet, China | **ON858914** | MT614336 | **ON890695** |
| I114_Isodon_muliensis | Y.P. Chen et al. EM701 (KUN) | Muli, Sichuan, China | **ON858915** | MT614329 | **ON890696** |
| I115_Isodon_flexicaulis | Y.P. Chen et al. EM673 (KUN) | Muli, Sichuan, China | **ON858916** | MT614338 | **ON890697** |
| I116_Isodon_yuennanensis | Y.P. Chen et al. EM602 (KUN) | Kunming, Yunnan, China | **ON858917** | **ON854744** | **ON890698** |
| I117_Isodon_setschwanensis | Y.P. Chen et al. EM646 (KUN) | Muli, Sichuan, China | **ON858918** | **ON854745** | **ON890699** |
| I118_Isodon_secundiflorus | Y.P. Chen et al. EM667 (KUN) | Muli, Sichuan, China | **ON858919** | MT614337 | **ON890700** |
| I119_Isodon_mucronatus | Y.P. Chen et al. EM676 (KUN) | Muli, Sichuan, China | **ON858920** | MT614328 | **ON890701** |
| I120_Isodon_medilungensis | Y.P. Chen et al. EM686 (KUN) | Muli, Sichuan, China | **ON858921** | **ON854747** | **ON890703** |
| I121_Isodon_chionanthus | Y.P. Chen et al. EM693 (KUN) | Muli, Sichuan, China | **ON858922** | **ON854748** | **ON890704** |
| I122_Isodon_bulleyanus | Y.P. Chen et al. EM596 (KUN) | Kunming, Yunnan, China | **ON858923** | **ON854749** | **ON890705** |
| I123_Isodon_phyllopodus | C.L. Xiang et al. XCL1353 (KUN) | Eryuan, Yunnan, China | **ON858924** | **ON854750** | **ON890706** |
| I124_Isodon_loxothyrsus | Y.P. Chen et al. EM703 (KUN) | Muli, Sichuan, China | **ON858925** | **ON854751** | **ON890707** |
| I127_Isodon_coetsa_var_coetsa | Y.P. Chen s.n. (KUN) | Kunming Botanical Garden (cultivated) | **ON858926** | **ON854752** | **ON890708** |
| I128_Isodon_schimperi | I. Friis & K. Vollesen 3783 (K) | Ethiopia | **ON858927** | **ON854753** | **ON890709** |
| I129_Isodon_ramosissimus | E. Phillips 3789B (K) | Mzimba, Malawi | **ON858928** | ON854754 | KF855442 |
| CU_Coleus_umbrosus | C.L. Xiang & Y.P. Chen s.n. (KUN) | Kew Botanical Gardens (cultivated) |  | **ON854734** | - |
| CX_Coleus_xanthanthus | - | - | MT473748 | MN116786 | MN116780 |
| HE_Hanceola_exserta | Y.P. Chen EM309 (KUN) | Hezhou, Guangxi, China | MT473765 | MN116783 | **ON890679** |
| HR_Hyptis_rhomboides | F. Zhao 30 (KUN) | Xishuangbanna Botanical Garden (cultivated) |  | **ON854735** | **ON890680** |
| HS_Hanceola_sinensis | Y.P. Chen et al. EM628 (KUN) | Emeishan, Sichuan, China |  | MN585134 | **ON890681** |
| LA_Lavandula_angustifolia | - | - | KY404093 | - | EF437225 |
| MS_Mesosphaerum_suaveolens | - | - | SRR6940078 | MN116784 | MN116777 |
| OB_Ocimum_basilicum | - | - | NC_035143 | KT210226 | DQ667240 |
| OI_Orthosiphon_incurvus | Q.R. Zhang et al. s.n. (KUN) | Nepal |  | **ON854795** | **ON890780** |
| PC_Platostoma_calcaratum | Z.J. Yin s.n. (KUN) | Mojiang, Yunnan, China |  | MH557900 | **ON890781** |
| PLC1_Plectranthus_cinereus | P. Kuchar 23573 (K) | Tanzania |  | **ON854796** | **ON890782** |
| PLC2_Plectranthus_cinereus | P.J. Greenway & Kanuari 11295 (K) | Tanzania |  | ON854797 | - |
| SF_Siphocranion_flavidum | C.L. Xiang et al. XCL1737 (KUN) | Malipo, Yunnan, China | MT473778 | MN585138 | **ON890783** |
| SM_Siphocranion_macranthum | Y.P. Chen et al. EM618 (KUN) | Nanchuan, Chongqing, China | MT473779 | MN585143 | **ON890784** |
| SN_Siphocranion_nudipes | Y.P. Chen et al. EM619 (KUN) | Nanchuan, Chongqing, China |  | MN585145 | **ON890785** |
| TN_Tetradenia_nervosa | C.L. Xiang & Y.P. Chen s.n. (KUN) | Kew Botanical Gardens (cultivated) |  | **ON854798** | **ON890786** |
