# Supplementary material for: Plastome sequences fail to resolve shallow level relationships within the rapidly radiated genus Isodon (Lamiaceae)
Source: Front Plant Sci. 2022 Sep 8;13:985488. doi: 10.3389/fpls.2022.985488 (PMC9493350; doi:10.3389/fpls.2022.985488)
Supplement: Supplementary file 8 [file Table_2.DOCX]

**TABLE S2.** Slope and *R^2^* values of the linear regressions between patristic and uncorrected pairwise distances for the maximum likelihood tree from each locus.

| **Treefile** | **Slope** | ***R^2^*** |
| --- | --- | --- |
| rpl22_rps19.tre | 0.256570624 | 0.428350964 |
| psbI_trnS_GCU.tre | 0.335418946 | 0.593849677 |
| infA_rps8.tre | 0.468923736 | 0.71395754 |
| rps4_trnT_UGU.tre | 0.485621224 | 0.948937641 |
| rrn23_trnA_UGC_exon2.tre | 0.498278094 | 0.741469884 |
| trnG_UCC_exon1_trnS_GCU.tre | 0.500864221 | 0.95365839 |
| psaA_ycf3_exon3.tre | 0.518016187 | 0.928839371 |
| rpoC2_rps2.tre | 0.532783202 | 0.871421469 |
| rps12.tre | 0.541823689 | 0.673389874 |
| atpE_trnM_CAU.tre | 0.54776464 | 0.843922938 |
| rpl36.tre | 0.55160638 | 0.849815108 |
| psbT.tre | 0.556250604 | 0.90282933 |
| rpl14_rps8.tre | 0.565192233 | 0.836716387 |
| atpA_atpF_exon2.tre | 0.573025814 | 0.952651611 |
| matK_trnK_UUU_exon2.tre | 0.605390644 | 0.957969259 |
| rps16_exon1_trnQ_UUG.tre | 0.606386315 | 0.980837511 |
| psaA_psaB.tre | 0.607138874 | 0.999997755 |
| ccsA_trnL_UAG.tre | 0.614450385 | 0.747645376 |
| psbK_trnQ_UUG.tre | 0.614876123 | 0.925561792 |
| psbA_trnH_GUG.tre | 0.624313162 | 0.869407902 |
| trnE_UUC_trnT_GGU.tre | 0.625219075 | 0.966879224 |
| ndhH_rps15.tre | 0.631636186 | 0.670033311 |
| ycf15_ycf2.tre | 0.631920757 | 0.67949582 |
| trnE_UUC_trnY_GUA.tre | 0.633944783 | 0.905082309 |
| trnG_UCC_exon2_trnR_UCU.tre | 0.645448529 | 0.918967185 |
| trnG_GCC_trnfM_CAU.tre | 0.646975837 | 0.935405939 |
| rps14_trnfM_CAU.tre | 0.647010333 | 0.639970127 |
| ndhG.tre | 0.67477888 | 0.9316189 |
| atpI_rps2.tre | 0.675116214 | 0.952041929 |
| petA_psbJ.tre | 0.686724862 | 0.958788994 |
| clpP_exon3_rps12_exon1.tre | 0.688070446 | 0.941816576 |
| ndhJ_trnF_GAA.tre | 0.688073925 | 0.94055091 |
| petN_psbM.tre | 0.690567699 | 0.874165314 |
| atpA_trnR_UCU.tre | 0.702882911 | 0.91705165 |
| psbB_psbT.tre | 0.704196096 | 0.979161422 |
| psaC.tre | 0.708831031 | 0.926380118 |
| psbN_psbT.tre | 0.713317106 | 0.948189601 |
| psbD_trnT_GGU.tre | 0.718033028 | 0.960228689 |
| petD_exon2_rpoA.tre | 0.724613357 | 0.936576868 |
| rbcL.tre | 0.742502276 | 0.980681106 |
| trnL_UAA_exon1_trnT_UGU.tre | 0.742641847 | 0.92040361 |
| psaJ_trnP_UGG.tre | 0.743484595 | 0.964546486 |
| ndhH.tre | 0.763352445 | 0.864808666 |
| petD_exon1_petD_exon2.tre | 0.766043691 | 0.966844064 |
| trnL_UAA_exon1_trnL_UAA_exon2.tre | 0.769922367 | 0.962725926 |
| rpl20_rps18.tre | 0.776800512 | 0.887168132 |
| rpl20_rps12_exon1.tre | 0.777673247 | 0.976181241 |
| ccsA_ndhD.tre | 0.779374327 | 0.975274423 |
| ndhF_rpl32.tre | 0.782704965 | 0.934057124 |
| accD_psaI.tre | 0.783748672 | 0.975549275 |
| atpH_atpI.tre | 0.790793284 | 0.990862859 |
| trnG_UCC_exon1_trnG_UCC_exon2.tre | 0.792292941 | 0.984504598 |
| ndhE_psaC.tre | 0.797438131 | 0.955882546 |
| psaJ_rpl33.tre | 0.79828014 | 0.985593111 |
| psbF.tre | 0.80045468 | 0.954344034 |
| psbZ_trnG_GCC.tre | 0.800882545 | 0.960854996 |
| rpoC1_exon1_rpoC1_exon2.tre | 0.802164599 | 0.989592313 |
| ndhG_ndhI.tre | 0.803965532 | 0.988738541 |
| cemA_ycf4.tre | 0.805073609 | 0.972682444 |
| trnS_GGA_ycf3_exon1.tre | 0.807166912 | 0.982705472 |
| petG_trnW_CCA.tre | 0.809042272 | 0.929039931 |
| petB_exon2_petD_exon1.tre | 0.809403005 | 0.975909751 |
| psaI_ycf4.tre | 0.809514342 | 0.940131198 |
| atpB_rbcL.tre | 0.81048333 | 0.986278179 |
| rps15_ycf1.tre | 0.810697261 | 0.979559965 |
| rpl32_trnL_UAG.tre | 0.811336158 | 0.981530723 |
| psbH.tre | 0.819200189 | 0.974191574 |
| petL_psbE.tre | 0.819249356 | 0.934977907 |
| rps19.tre | 0.832477944 | 0.988832164 |
| petD.tre | 0.832747366 | 0.948560433 |
| clpP_exon2_clpP_exon3.tre | 0.839101158 | 0.991872268 |
| ndhC_trnV_UAC_exon2.tre | 0.84935256 | 0.990182213 |
| ycf1.tre | 0.85490916 | 0.997306582 |
| petB.tre | 0.855874977 | 0.973644678 |
| rpoA_rps11.tre | 0.85893151 | 0.965055596 |
| rps11.tre | 0.862288006 | 0.994287487 |
| psbI_psbK.tre | 0.863124362 | 0.952258082 |
| psbJ_psbL.tre | 0.865438307 | 0.966978339 |
| rps16_exon2_trnK_UUU_exon1.tre | 0.865640894 | 0.988141103 |
| atpF_exon1_atpF_exon2.tre | 0.869424585 | 0.994011897 |
| cemA_petA.tre | 0.870086652 | 0.964711838 |
| ndhE_ndhG.tre | 0.87076881 | 0.990284142 |
| rpoB_trnC_GCA.tre | 0.872580153 | 0.9905367 |
| rps4_trnS_GGA.tre | 0.876932679 | 0.962144116 |
| clpP.tre | 0.877108291 | 0.992759092 |
| rpoC1_exon2_rpoC2.tre | 0.878428837 | 0.954915053 |
| accD_rbcL.tre | 0.882279903 | 0.983067147 |
| ndhD.tre | 0.886580714 | 0.991418229 |
| ndhA_exon1_ndhA_exon2.tre | 0.887045134 | 0.989507185 |
| petB_exon1_petB_exon2.tre | 0.889745813 | 0.995526363 |
| petB_exon1_psbH.tre | 0.889848373 | 0.968594831 |
| petN_trnC_GCA.tre | 0.889964635 | 0.972763135 |
| ndhD_psaC.tre | 0.890303408 | 0.884850617 |
| rpl16_exon1_rpl16_exon2.tre | 0.891584589 | 0.987190457 |
| rpl2_exon2_rps19.tre | 0.897111444 | 0.989805623 |
| clpP_exon1_psbB.tre | 0.89867647 | 0.98715326 |
| ndhE.tre | 0.898805525 | 0.991621063 |
| trnI_GAU_exon1_trnI_GAU_exon2.tre | 0.898885578 | 0.935129221 |
| petG_petL.tre | 0.901229899 | 0.840988401 |
| ndhB_exon2_trnL_CAA.tre | 0.901467915 | 0.987321872 |
| trnP_UGG_trnW_CCA.tre | 0.903952469 | 0.980892504 |
| trnF_GAA_trnL_UAA_exon2.tre | 0.904788922 | 0.993768413 |
| matK.tre | 0.904827732 | 0.994985081 |
| matK_trnK_UUU_exon1.tre | 0.906961041 | 0.996432003 |
| rpl33_rps18.tre | 0.90719625 | 0.949957042 |
| ccsA.tre | 0.911512994 | 0.990185289 |
| rps15.tre | 0.912421778 | 0.972020335 |
| psbA.tre | 0.912611665 | 0.979913351 |
| rps16_exon1_rps16_exon2.tre | 0.915110159 | 0.987864063 |
| rpl36_rps11.tre | 0.915378375 | 0.945010917 |
| rpl14_rpl16_exon2.tre | 0.916289864 | 0.98538732 |
| ycf4.tre | 0.917452047 | 0.986556818 |
| ndhF.tre | 0.917577691 | 0.994123225 |
| ndhC.tre | 0.919120987 | 0.985812002 |
| ndhI.tre | 0.919495932 | 0.986313905 |
| trnN_GUU_ycf1.tre | 0.920020391 | 0.978892381 |
| psbI.tre | 0.92074208 | 0.995725997 |
| clpP_exon1_clpP_exon2.tre | 0.922444287 | 0.989638001 |
| rps12_exon3_rps7.tre | 0.923484686 | 0.999997795 |
| rps16.tre | 0.924098131 | 0.985744575 |
| psbZ_trnS_UGA.tre | 0.925880945 | 0.992749377 |
| rpl14.tre | 0.926594128 | 0.987556563 |
| ndhJ.tre | 0.928852236 | 0.991882193 |
| trnD_GUC_trnY_GUA.tre | 0.931282121 | 0.992258558 |
| rrn4.5_rrn5.tre | 0.933999508 | 0.993434533 |
| rps3.tre | 0.936916833 | 0.991606065 |
| ycf3_exon2_ycf3_exon3.tre | 0.938065625 | 0.992444486 |
| rpl16_exon1_rps3.tre | 0.939369523 | 0.972965035 |
| atpE.tre | 0.940095409 | 0.989573838 |
| rpl22.tre | 0.941273791 | 0.987641747 |
| trnA_UGC_exon1_trnI_GAU_exon2.tre | 0.94531341 | 0.997322918 |
| rps12_exon2_trnV_GAC.tre | 0.945464174 | 0.977953993 |
| trnV_UAC_exon1_trnV_UAC_exon2.tre | 0.94554875 | 0.992014246 |
| ndhJ_ndhK.tre | 0.946628667 | 0.989045386 |
| psbC.tre | 0.947308572 | 0.990462294 |
| petG.tre | 0.947320604 | 0.985082404 |
| atpB.tre | 0.947866267 | 0.99306936 |
| psbD.tre | 0.950709608 | 0.992946848 |
| trnM_CAU_trnV_UAC_exon1.tre | 0.950895023 | 0.989879268 |
| infA_rpl36.tre | 0.951323515 | 0.980116661 |
| psbB.tre | 0.951698711 | 0.996061862 |
| petA.tre | 0.952256349 | 0.9899796 |
| psbC_trnS_UGA.tre | 0.956033259 | 0.994813158 |
| ycf2.tre | 0.956227635 | 0.994480401 |
| trnL_CAA_ycf15.tre | 0.958620953 | 0.99027042 |
| atpA.tre | 0.95920105 | 0.997666443 |
| psbM_trnD_GUC.tre | 0.95952064 | 0.995672542 |
| rrn5_trnR_ACG.tre | 0.960959525 | 0.98936306 |
| psbN.tre | 0.961298032 | 0.999071568 |
| rpoA.tre | 0.961593373 | 0.99759831 |
| trnI_CAU_ycf2.tre | 0.961678377 | 0.999903449 |
| ycf3_exon1_ycf3_exon2.tre | 0.962101233 | 0.995262103 |
| psaJ.tre | 0.962213584 | 0.996057054 |
| atpF_exon1_atpH.tre | 0.962768566 | 0.999145367 |
| rpoB_rpoC1_exon1.tre | 0.963109487 | 0.999957371 |
| psaB.tre | 0.964589772 | 0.996488297 |
| trnN_GUU_trnR_ACG.tre | 0.964902692 | 0.994435797 |
| rpoC2.tre | 0.965823895 | 0.994509847 |
| ndhK.tre | 0.96610649 | 0.995863139 |
| ndhC_ndhK.tre | 0.967409786 | 0.999876469 |
| accD.tre | 0.967485571 | 0.997230668 |
| psaA.tre | 0.96833237 | 0.99792513 |
| ycf15.tre | 0.96912608 | 0.992423017 |
| cemA.tre | 0.96979869 | 0.997543417 |
| rrn23_rrn4.5.tre | 0.970801554 | 0.999823018 |
| ndhA_exon2_ndhI.tre | 0.97365065 | 0.997217558 |
| ndhA.tre | 0.973652507 | 0.997938211 |
| psbK.tre | 0.974717975 | 0.996541787 |
| rpoC1.tre | 0.974915389 | 0.999312081 |
| psbL.tre | 0.975419815 | 0.999962877 |
| rps18.tre | 0.975996965 | 0.996924464 |
| atpI.tre | 0.976766732 | 0.996634698 |
| rpl2.tre | 0.976864643 | 0.986394026 |
| ndhB_exon1_ndhB_exon2.tre | 0.977244124 | 0.995087049 |
| psbH_psbN.tre | 0.978345739 | 0.999638723 |
| psbA_trnK_UUU_exon2.tre | 0.978958306 | 0.997847622 |
| rps4.tre | 0.97919398 | 0.99473472 |
| rps12_exon2_rps12_exon3.tre | 0.979343547 | 0.997844618 |
| rpoB.tre | 0.980112228 | 0.99906139 |
| rpl23_trnI_CAU.tre | 0.982238499 | 0.999696245 |
| rrn16_trnV_GAC.tre | 0.982766867 | 0.983778807 |
| atpF.tre | 0.98326003 | 0.998146623 |
| infA.tre | 0.98394797 | 0.996926743 |
| psaB_rps14.tre | 0.985549757 | 0.999904013 |
| ndhB_exon1_rps7.tre | 0.98559404 | 0.999578455 |
| rpl16.tre | 0.986253777 | 0.999323862 |
| petN.tre | 0.986326234 | 0.999940392 |
| rps8.tre | 0.986968551 | 0.997595721 |
| psbM.tre | 0.986988825 | 0.999960585 |
| rpl2_exon1_rpl2_exon2.tre | 0.987385123 | 0.999599574 |
| psbE.tre | 0.990715822 | 0.999705482 |
| psaI.tre | 0.992012821 | 0.999974243 |
| ycf3.tre | 0.993507608 | 0.999704795 |
| psbJ.tre | 0.994350204 | 0.999923307 |
| rps14.tre | 0.994531154 | 0.999989168 |
| rps2.tre | 0.994950718 | 0.999545832 |
| rps7.tre | 0.995260768 | 0.999665879 |
| psbZ.tre | 0.995574607 | 0.999964466 |
| rrn16_trnI_GAU_exon1.tre | 0.995625544 | 0.999966634 |
| trnA_UGC_exon1_trnA_UGC_exon2.tre | 0.996019465 | 0.99996043 |
| ndhB.tre | 0.996896592 | 0.999945384 |
| atpH.tre | 0.997220115 | 0.999979577 |
| rpl32.tre | 0.997660213 | 0.998016009 |
| rpl23.tre | 0.997988425 | 0.999081631 |
| petL.tre | 1.003973553 | 0.995534569 |
| rpl33.tre | 1.009327006 | 0.998561732 |
| rpl20.tre | 1.010360059 | 0.992100407 |
