# Supplementary material for: Plastome sequences fail to resolve shallow level relationships within the rapidly radiated genus Isodon (Lamiaceae)
Source: Front Plant Sci. 2022 Sep 8;13:985488. doi: 10.3389/fpls.2022.985488 (PMC9493350; doi:10.3389/fpls.2022.985488)
Supplement: Supplementary file 9 [file Table_3.DOCX]

**TABLE S3.** Features of plastomes of *Isodon* species. LSC: large single copy; SSC: small single copy; IR: inverted repeat.

| **Code** | **Species** | **Nucleatide length [bp]** | | | | **Number of genes** | | | **GC%** | | | |
| --- | --- | --- | --- | --- | --- | --- | --- | --- | --- | --- | --- | --- |
|  |  | **Total** | **LSC** | **SSC** | **IR** | **Protein** | **tRNA** | **rRNA** | **Total** | **LSC** | **SSC** | **IR** |
| I1 | Isodon adenanthus | 152,602 | 83,583 | 17,559 | 25,730 | 80 | 30 | 4 | 37.70% | 35.60% | 31.10% | 43.10% |
| I2 | Isodon albopilosus | 152,701 | 83,570 | 17,679 | 25,726 | 80 | 30 | 4 | 37.60% | 35.60% | 31.00% | 43.10% |
| I3 | Isodon alborubrus | 152,709 | 83,580 | 17,677 | 25,726 | 80 | 30 | 4 | 37.60% | 35.60% | 31.00% | 43.10% |
| I4 | Isodon amethystoides | 152,639 | 83,519 | 17,670 | 25,725 | 80 | 30 | 4 | 37.60% | 35.70% | 31.10% | 43.10% |
| I5 | Isodon angustifolius | 152,694 | 83,575 | 17,667 | 25,726 | 80 | 30 | 4 | 37.60% | 35.60% | 31.00% | 43.10% |
| I6 | Isodon anisochilus | 152,714 | 83,603 | 17,661 | 25,725 | 80 | 30 | 4 | 37.60% | 35.60% | 31.00% | 43.10% |
| I7 | Isodon atroruber | 152,187 | 83,088 | 17,699 | 25,700 | 80 | 30 | 4 | 37.60% | 35.60% | 31.00% | 43.10% |
| I8 | Isodon aurantiacus | 152,693 | 83,571 | 17,670 | 25,726 | 80 | 30 | 4 | 37.60% | 35.70% | 31.00% | 43.10% |
| I9 | Isodon barbeyanus | 152,654 | 83,524 | 17,678 | 25,726 | 80 | 30 | 4 | 37.60% | 35.60% | 31.00% | 43.10% |
| I10 | Isodon brachythyrsus | 152,697 | 83,577 | 17,666 | 25,727 | 80 | 30 | 4 | 37.60% | 35.60% | 31.00% | 43.10% |
| I11 | Isodon bulleyanus | 152,645 | 83,528 | 17,665 | 25,726 | 80 | 30 | 4 | 37.60% | 35.60% | 31.00% | 43.10% |
| I12 | Isodon calcicolus | 152,594 | 83,486 | 17,666 | 25,721 | 80 | 30 | 4 | 37.60% | 35.60% | 31.00% | 43.10% |
| I13 | Isodon coetsa var. coetsa | 152,745 | 83,602 | 17,687 | 25,728 | 80 | 30 | 4 | 37.60% | 35.60% | 31.10% | 43.10% |
| I14 | Isodon coetsa var. cavaleriei | 152,674 | 83,559 | 17,663 | 25,726 | 80 | 30 | 4 | 37.60% | 35.60% | 31.00% | 43.10% |
| I15 | Isodon coetsoides | 152,692 | 83,549 | 17,687 | 25,728 | 80 | 30 | 4 | 37.60% | 35.60% | 31.10% | 43.10% |
| I16 | Isodon dawoensis | 152,709 | 83,599 | 17,656 | 25,727 | 80 | 30 | 4 | 37.60% | 35.60% | 31.00% | 43.10% |
| I17 | Isodon delavayi | 152,661 | 83,543 | 17,670 | 25,724 | 80 | 30 | 4 | 37.60% | 35.60% | 31.00% | 43.10% |
| I18 | Isodon enanderianus | 152,680 | 83,567 | 17,661 | 25,726 | 80 | 30 | 4 | 37.60% | 35.60% | 31.00% | 43.10% |
| I19 | Isodon eriocalyx | 152,629 | 83,506 | 17,671 | 25,726 | 80 | 30 | 4 | 37.60% | 35.70% | 31.00% | 43.10% |
| I21 | Isodon excisoides | 152,646 | 83,531 | 17,663 | 25,726 | 80 | 30 | 4 | 37.60% | 35.60% | 31.00% | 43.10% |
| I22 | Isodon excisus | 152,290 | 83,184 | 17,654 | 25,726 | 80 | 30 | 4 | 37.60% | 35.70% | 31.10% | 43.10% |
| I24 | Isodon flavidus | 152,257 | 83,215 | 17,698 | 25,672 | 80 | 30 | 4 | 37.60% | 35.60% | 31.10% | 43.10% |
| I25 | Isodon flavidus | 152,266 | 83,231 | 17,691 | 25,672 | 80 | 30 | 4 | 37.60% | 35.60% | 31.00% | 43.10% |
| I26 | Isodon forrestii | 152,643 | 83,521 | 17,670 | 25,726 | 80 | 30 | 4 | 37.60% | 35.60% | 31.00% | 43.10% |
| I27 | Isodon gibbosus | 152,720 | 83,611 | 17,659 | 25,725 | 80 | 30 | 4 | 37.60% | 35.60% | 31.00% | 43.10% |
| I29 | Isodon grandifolius var. atuntzeensis | 152,693 | 83,573 | 17,668 | 25,726 | 80 | 30 | 4 | 37.60% | 35.60% | 31.00% | 43.10% |
| I30 | Isodon hirtellus | 152,623 | 83,502 | 17,669 | 25,726 | 80 | 30 | 4 | 37.60% | 35.60% | 31.00% | 43.10% |
| I33 | Isodon hsiwenii | 152,633 | 83,527 | 17,652 | 25,727 | 80 | 30 | 4 | 37.60% | 35.60% | 31.10% | 43.10% |
| I34 | Isodon inflexus | 152,606 | 83,495 | 17,659 | 25,726 | 80 | 30 | 4 | 37.60% | 35.70% | 31.00% | 43.10% |
| I35 | Isodon interruptus | 152,652 | 83,529 | 17,671 | 25,726 | 80 | 30 | 4 | 37.60% | 35.60% | 31.00% | 43.10% |
| I36 | Isodon irroratus | 152,688 | 83,564 | 17,672 | 25,726 | 80 | 30 | 4 | 37.60% | 35.70% | 31.10% | 43.10% |
| I37 | Isodon irroratus | 152,694 | 83,575 | 17,667 | 25,726 | 80 | 30 | 4 | 37.60% | 35.60% | 31.00% | 43.10% |
| I38 | Isodon irroratus | 152,681 | 83,567 | 17,662 | 25,726 | 80 | 30 | 4 | 37.60% | 35.60% | 31.10% | 43.10% |
| I39 | Isodon japonicus var. glaucocalyx | 152,660 | 83,561 | 17,655 | 25,722 | 80 | 30 | 4 | 37.60% | 35.70% | 31.10% | 43.10% |
| I40 | Isodon japonicus var. japonicus | 152,674 | 83,568 | 17,662 | 25,722 | 80 | 30 | 4 | 37.60% | 35.60% | 31.10% | 43.10% |
| I41 | Isodon kangtingensis | 152,299 | 83,184 | 17,663 | 25,726 | 80 | 30 | 4 | 37.60% | 35.70% | 31.00% | 43.10% |
| I42 | Isodon leucophyllus | 152,693 | 83,571 | 17,670 | 25,726 | 80 | 30 | 4 | 37.60% | 35.60% | 31.00% | 43.10% |
| I43 | Isodon grandifolius var. grandifolius | 152,577 | 83,486 | 17,667 | 25,712 | 80 | 30 | 4 | 37.60% | 35.60% | 31.00% | 43.10% |
| I44 | Isodon macrocalyx | 152,297 | 83,183 | 17,662 | 25,726 | 80 | 30 | 4 | 37.60% | 35.60% | 31.10% | 43.10% |
| I45 | Isodon lophanthoides var. graciliflorus | 152,208 | 83,079 | 17,729 | 25,700 | 80 | 30 | 4 | 37.60% | 35.60% | 31.00% | 43.10% |
| I47 | Isodon lophanthoides var. lophanthoides | 152,195 | 83,096 | 17,699 | 25,700 | 80 | 30 | 4 | 37.60% | 35.60% | 31.00% | 43.10% |
| I48 | Isodon loxothyrsus | 152,640 | 83,518 | 17,670 | 25,726 | 80 | 30 | 4 | 37.60% | 35.60% | 31.00% | 43.10% |
| I49 | Isodon lungshengensis | 152,253 | 83,139 | 17,662 | 25,726 | 80 | 30 | 4 | 37.60% | 35.70% | 31.10% | 43.10% |
| I50 | Isodon macrophyllus | 152,596 | 83,466 | 17,674 | 25,728 | 80 | 30 | 4 | 37.60% | 35.60% | 31.00% | 43.10% |
| I51 | Isodon bifidocalyx | 152,285 | 83,170 | 17,663 | 25,726 | 80 | 30 | 4 | 37.60% | 35.70% | 31.00% | 43.10% |
| I52 | Isodon megathyrsus | 152,568 | 83,584 | 17,532 | 25,726 | 80 | 30 | 4 | 37.60% | 35.60% | 31.10% | 43.10% |
| I53 | Isodon megathyrsus | 152,298 | 83,184 | 17,662 | 25,726 | 80 | 30 | 4 | 37.60% | 35.60% | 31.10% | 43.10% |
| I54 | Isodon nervosus | 152,291 | 83,177 | 17,662 | 25,726 | 80 | 30 | 4 | 37.60% | 35.70% | 31.10% | 43.10% |
| I56 | Isodon oreophilus | 152,274 | 83,232 | 17,698 | 25,672 | 80 | 30 | 4 | 37.60% | 35.60% | 31.00% | 43.10% |
| I57 | Isodon oresbius | 152,626 | 83,499 | 17,675 | 25,726 | 80 | 30 | 4 | 37.60% | 35.60% | 31.00% | 43.10% |
| I58 | Isodon parvifolius | 152,713 | 83,604 | 17,657 | 25,726 | 80 | 30 | 4 | 37.60% | 35.60% | 31.00% | 43.10% |
| I59 | Isodon parvifolius | 152,681 | 83,571 | 17,656 | 25,727 | 80 | 30 | 4 | 37.60% | 35.60% | 31.00% | 43.10% |
| I60 | Isodon pesudo-irroratus | 152,698 | 83,591 | 17,655 | 25,726 | 80 | 30 | 4 | 37.60% | 35.60% | 31.00% | 43.10% |
| I61 | Isodon pharicus | 152,684 | 83,574 | 17,656 | 25,727 | 80 | 30 | 4 | 37.70% | 35.70% | 31.10% | 43.10% |
| I63 | Isodon phyllostachys | 152,699 | 83,584 | 17,661 | 25,727 | 80 | 30 | 4 | 37.60% | 35.60% | 31.00% | 43.10% |
| I64 | Isodon pleiophyllus | 152,651 | 83,533 | 17,670 | 25,724 | 80 | 30 | 4 | 37.60% | 35.60% | 31.00% | 43.10% |
| I69 | Isodon rubescens | 152,651 | 83,567 | 17,640 | 25,722 | 80 | 30 | 4 | 37.60% | 35.60% | 31.10% | 43.10% |
| I70 | Isodon rugosiformis | 152,650 | 83,529 | 17,669 | 25,726 | 80 | 30 | 4 | 37.60% | 35.70% | 31.00% | 43.10% |
| I71 | Isodon rugosus | 152,677 | 83,554 | 17,669 | 25,727 | 80 | 30 | 4 | 37.60% | 35.70% | 31.00% | 43.10% |
| I72 | Isodon scoparius | 152,610 | 83,513 | 17,657 | 25,720 | 80 | 30 | 4 | 37.60% | 35.60% | 31.00% | 43.10% |
| I73 | Isodon scrophularioides | 152,268 | 83,178 | 17,690 | 25,700 | 80 | 30 | 4 | 37.60% | 35.60% | 31.00% | 43.10% |
| I74 | Isodon sculponeatus | 152,298 | 83,184 | 17,662 | 25,726 | 80 | 30 | 4 | 37.60% | 35.60% | 31.10% | 43.10% |
| I75 | Isodon serra | 152,551 | 83,434 | 17,661 | 25,728 | 80 | 30 | 4 | 37.60% | 35.70% | 31.10% | 43.10% |
| I77 | Isodon smithianus | 152,672 | 83,555 | 17,667 | 25,725 | 80 | 30 | 4 | 37.60% | 35.70% | 31.00% | 43.10% |
| I79 | Isodon tenuifolius | 152,656 | 83,534 | 17,670 | 25,726 | 80 | 30 | 4 | 37.60% | 35.60% | 31.00% | 43.10% |
| I80 | Isodon ternifolius | 152,824 | 83,640 | 17,702 | 25,741 | 80 | 30 | 4 | 37.60% | 35.70% | 31.10% | 43.10% |
| I81 | Isodon villosus | 152,172 | 83,073 | 17,699 | 25,700 | 80 | 30 | 4 | 37.60% | 35.60% | 31.00% | 43.10% |
| I83 | Isodon wardii | 152,678 | 83,553 | 17,673 | 25,726 | 80 | 30 | 4 | 37.60% | 35.70% | 31.10% | 43.10% |
| I84 | Isodon weisiensis | 152,693 | 83,572 | 17,669 | 25,726 | 80 | 30 | 4 | 37.60% | 35.70% | 31.00% | 43.10% |
| I86 | Isodon wikstroemioides | 152,652 | 83,530 | 17,670 | 25,726 | 80 | 30 | 4 | 37.60% | 35.60% | 31.00% | 43.10% |
| I88 | Isodon yuennanensis | 151,923 | 82,906 | 17,673 | 25,672 | 80 | 30 | 4 | 37.60% | 35.60% | 31.00% | 43.10% |
| I89 | Isodon xerophilus | 152,466 | 83,349 | 17,665 | 25,726 | 80 | 30 | 4 | 37.60% | 35.70% | 31.00% | 43.10% |
| I98 | Isodon ternifolius | 152,820 | 83,636 | 17,702 | 25,741 | 80 | 30 | 4 | 37.60% | 35.70% | 31.10% | 43.10% |
| I99 | Isodon japonicus var. japonicus | 152,684 | 83,544 | 17,676 | 25,732 | 80 | 30 | 4 | 37.60% | 35.60% | 31.00% | 43.10% |
| I100 | Isodon trichocarpus | 152,299 | 83,185 | 17,662 | 25,726 | 80 | 30 | 4 | 37.60% | 35.70% | 31.10% | 43.10% |
| I101 | Isodon effusus | 152,298 | 83,184 | 17,662 | 25,726 | 80 | 30 | 4 | 37.60% | 35.70% | 31.10% | 43.10% |
| I102 | Isodon umbrosus var. leucanthus | 152,682 | 83,564 | 17,662 | 25,728 | 80 | 30 | 4 | 37.60% | 35.70% | 31.00% | 43.10% |
| I103 | Isodon longitubus | 152,660 | 83,545 | 17,661 | 25,727 | 80 | 30 | 4 | 37.60% | 35.60% | 31.00% | 43.10% |
| I104 | Isodon shikokianus var. occidentalis | 152,677 | 83,561 | 17,660 | 25,728 | 80 | 30 | 4 | 37.60% | 35.70% | 31.10% | 43.10% |
| I107 | Isodon excisoides | 152,600 | 83,540 | 17,670 | 25,695 | 80 | 30 | 4 | 37.60% | 35.60% | 31.00% | 43.10% |
| I108 | Isodon flabelliformis | 152,298 | 83,184 | 17,662 | 25,726 | 80 | 30 | 4 | 37.60% | 35.70% | 31.10% | 43.10% |
| I110 | Isodon polystachys | 152,681 | 83,571 | 17,656 | 25,727 | 80 | 30 | 4 | 37.60% | 35.60% | 31.00% | 43.10% |
| I111 | Isodon racemosus | 152,683 | 83,568 | 17,671 | 25,722 | 80 | 30 | 4 | 37.60% | 35.70% | 31.10% | 43.10% |
| I112 | Isodon rosthornii | 152,322 | 83,219 | 17,651 | 25,726 | 80 | 30 | 4 | 37.60% | 35.70% | 31.10% | 43.10% |
| I113 | Isodon rugosus | 152,763 | 83,539 | 17,706 | 25,759 | 80 | 30 | 4 | 37.60% | 35.60% | 31.10% | 43.10% |
| I114 | Isodon muliensis | 152,695 | 83,553 | 17,690 | 25,726 | 80 | 30 | 4 | 37.60% | 35.70% | 31.00% | 43.10% |
| I115 | Isodon flexicaulis | 152,662 | 83,555 | 17,655 | 25,726 | 80 | 30 | 4 | 37.60% | 35.60% | 31.10% | 43.10% |
| I116 | Isodon yuennanensis | 152,265 | 83,230 | 17,691 | 25,672 | 80 | 30 | 4 | 37.60% | 35.60% | 31.00% | 43.10% |
| I117 | Isodon setschwanensis | 152,669 | 83,535 | 17,682 | 25,726 | 80 | 30 | 4 | 37.60% | 35.60% | 31.00% | 43.10% |
| I118 | Isodon secundiflorus | 152,680 | 83,566 | 17,662 | 25,726 | 80 | 30 | 4 | 37.60% | 35.60% | 31.00% | 43.10% |
| I119 | Isodon mucronatus | 152,637 | 83,520 | 17,665 | 25,726 | 80 | 30 | 4 | 37.60% | 35.60% | 31.00% | 43.10% |
| I120 | Isodon medilungensis | 152,714 | 83,600 | 17,662 | 25,726 | 80 | 30 | 4 | 37.60% | 35.60% | 31.10% | 43.10% |
| I121 | Isodon chionanthus | 152,498 | 83,377 | 17,669 | 25,726 | 80 | 30 | 4 | 37.60% | 35.70% | 31.00% | 43.10% |
| I122 | Isodon bulleyanus | 152,281 | 83,168 | 17,661 | 25,726 | 80 | 30 | 4 | 37.60% | 35.70% | 31.10% | 43.10% |
| I123 | Isodon phyllopodus | 152,240 | 83,207 | 17,691 | 25,671 | 80 | 30 | 4 | 37.60% | 35.60% | 31.10% | 43.10% |
| I124 | Isodon loxothyrsus | 152,653 | 83,552 | 17,673 | 25,714 | 80 | 30 | 4 | 37.60% | 35.70% | 31.00% | 43.10% |
| I127 | Isodon coetsa var. coetsa | 152,658 | 83,544 | 17,662 | 25,726 | 80 | 30 | 4 | 37.60% | 35.60% | 31.00% | 43.10% |
| I128 | Isodon schimperi | 152,450 | 83,294 | 17,676 | 25,740 | 80 | 30 | 4 | 37.60% | 35.60% | 31.00% | 43.10% |
| I129 | Isodon ramosissimus | 152,473 | 83,315 | 17,684 | 25,737 | 80 | 30 | 4 | 37.50% | 35.50% | 30.90% | 43.10% |
