# Supplementary material for: Plastome sequences fail to resolve shallow level relationships within the rapidly radiated genus Isodon (Lamiaceae)
Source: Front Plant Sci. 2022 Sep 8;13:985488. doi: 10.3389/fpls.2022.985488 (PMC9493350; doi:10.3389/fpls.2022.985488)
Supplement: Supplementary file 10 [file Table_4.DOCX]

**TABLE S4.** Nutlet morphology of *Isodon* species sampled in present study. Herbarium acronyms are listed in bracts after vouchers.

| **Taxon** | **Voucher** | **Location** | **Length (mm)** | **Width (mm)** | **Length/Width** | **Surface type** |
| --- | --- | --- | --- | --- | --- | --- |
| **Clade I** | | | | | | |
| *Isodon atroruber* | G.X. Hu et al. 1209005 (KUN) | Cona, Tibet, China | 1.19±0.05 | 0.8±0.06 | 1.5±0.09 | Psilate |
| *I. lophanthoides* var. *lophanthoides* | H. Peng et al. HP5733 (KUN) | Menghai, Yunnan, China | 0.89±0.06 | 0.58±0.02 | 1.55±0.1 | Psilate |
| *I. lophanthoides* var. *graciliflorus* | Y.P. Chen et al. EM279 (KUN) | Guilin, Guangxi, China | 0.91±0.06 | 0.67±0.05 | 1.36±0.07 | Psilate |
| *I. oreopilus* | E.D. Liu et al. 2702 (KUN) | Shangri-La, Yunnan, China | 1.07±0.08 | 0.91±0.05 | 1.17±0.11 | Reticulate |
| *I. phyllopodus* | H.J. Dong et al. D406 (KUN) | Dali, Yunnan, China | 1.06±0.04 | 0.82±0.03 | 1.29±0.06 | Reticulate |
| *I. scrophularioides* | G.X. Hu et al. 1209013 (KUN) | Cona, Tibet, China | 1.19±0.04 | 0.99±0.06 | 1.2±0.07 | Reticulate |
| *I. villosus* | Y.P. Chen EM227 (KUN) | Hezhou, Guangxi, China | 1.4±0.07 | 0.94±0.05 | 1.49±0.07 | Psilate |
| **Clade II** | | | | | | |
| *I. ramosissimus* | Perdue & Kibuwa 9300 (K) | Africa | 0.90±0.05 | 0.68±0.03 | 1.33±0.05 | Reticulate-papillate |
| *I. schimperi* | F.G. Meyer 8981 (K) | Ethiopia | 1.34±0.03 | 0.90±0.01 | 1.49±0.01 | Reticulate-papillate |
| **Clade III** | | | | | | |
| *I. ternifolius* | H.T. Tsai 55512（KUN） | Longling, Yunnan, China | 1.62±0.06 | 0.66±0.04 | 2.48±0.14 | Striate |
| **Clade IV** | | | | | | |
| *I. adenanthus* | Y.P. Chen et al. XS-04 (KUN) | Kunming, Yunnan, China | 1.32±0.12 | 1.21±0.06 | 1.09±0.07 | Cellular |
| *I. albopilosus* | E.D. Liu et al.3125 (KUN) | Huixian, Gansu, China | 1.53±0.07 | 1.24±0.08 | 1.24±0.08 | Cellular |
| *I. alborubrus* | H.J. Dong 1011 (KUN) | Shiping, Yunnan, China | 1.48±0.08 | 1.04±0.08 | 1.42±0.06 | Cellular |
| *I. amethystoides* | Y.P. Chen et al. EM283 (KUN) | Guilin, Guangxi, China | 1.51±0.09 | 1.17±0.09 | 1.29±0.08 | Glandular |
| *I. angustifolius* | Y.P. Chen et al. EM135 (KUN) | Yongsheng, Yunnan, China | 1.58±0.08 | 1.27±0.1 | 1.25±0.09 | Cellular |
| *I. anisochilus* | E.D. Liu et al. 2906 (KUN) | Muli, Sichuan, China | 1.51±0.08 | 1.07±0.1 | 1.43±0.2 | Cellular |
| *I. aurantiacus* | G.X. Hu et al. 1209054 (KUN) | Nang, Tibet, China | 1.44±0.09 | 1.04±0.09 | 1.39±0.08 | Cellular |
| *I. barbeyanus* | E.D. Liu et al. 3208 (KUN) | Daofu, Sichuan, China | 1.53±0.08 | 0.99±0.07 | 1.55±0.11 | Cellular |
| *I. bifidocalyx* | Y.P. Chen et al. EM040 (KUN) | Lin'an, Zhejiang, China | 1.58±0.09 | 1.28±0.1 | 1.24±0.07 | Glandular |
| *I. bulleyanus* | H.J. Dong et al. D402 (KUN) | Dali, Yunnan, China | 1.75±0.07 | 1.13±0.12 | 1.57±0.19 | Cellular |
| *I. coetsa* var. *coetsa* | Y.P. Chen et al. EM162 (KUN) | Longling, Yunnan, China | 1.07±0.04 | 0.83±0.05 | 1.29±0.06 | Cellular |
| *I. coetsa* var. *cavaleriei* | Y.P. Chen et al. EM241 (KUN) | Qiaojia, Yunnan, China | 1.18±0.07 | 0.79±0.05 | 1.5±0.11 | Cellular |
| *I. dawoensis* | E.D. Liu et al.3226 (KUN) | Luhuo, Sichuan, China | 1.64±0.09 | 1.13±0.08 | 1.46±0.06 | Cellular |
| *I. delavayi* | C.L. Xiang et al. 540 (KUN) | Eryuan, Yunnan, China | 1.52±0.09 | 1.1±0.03 | 1.39±0.06 | Cellular |
| *I. enanderianus* | Y.P. Chen et al. SD254 (KUN) | Shiping, Yunnan, China | 0.93±0.03 | 0.76±0.04 | 1.23±0.08 | Cellular |
| *I. eriocalyx* | Y.P. Chen et al. EM246 (KUN) | Daguan, Yunnan, China | 1.03±0.05 | 0.75±0.03 | 1.39±0.09 | Cellular |
| *I. excisus* | Y.P. Chen et al. EM215 (KUN) | Antu, Jilin, China | 1.55±0.08 | 1.34±0.09 | 1.16±0.08 | Glandular |
| *I. forrestii* | Y.P. Chen et al. 2012-366 (KUN) | Lijiang, Yunnan, China | 1.56±0.09 | 1.18±0.1 | 1.33±0.13 | Cellular |
| *I. gibbosus* | Y.P. Chen et al. EM281 (KUN) | Guilin, Guangxi, China | 1.44±0.06 | 1.09±0.07 | 1.33±0.09 | Cellular |
| *I. grandifolius* var. *atuntzeensis* | E.D. Liu et al. 3254 (KUN) | Jomda, Tibet, China | 1.37±0.05 | 0.94±0.07 | 1.47±0.13 | Cellular |
| *I. hirtellus* | Y.P. Chen et al. EM107 (KUN) | Dongchuan, Yunnan, China | 1.13±0.09 | 0.79±0.07 | 1.44±0.09 | Cellular |
| *I. interruptus* | Y.P. Chen et al. EM295 (KUN) | Kunming, Yunnan, China | 1.13±0.06 | 0.79±0.05 | 1.44±0.11 | Cellular |
| *I. irroratus* | H.J. Dong et al. D495 (KUN) | Lijiang, Yunnan, China | 1.87±0.07 | 1.38±0.12 | 1.36±0.1 | Cellular |
| *I. japonicus* var. *glaucocalyx* | Y.P. Chen et al. EM030 (KUN) | Fuyuan, Heilongjiang, China | 1.14±0.06 | 0.86±0.04 | 1.32±0.07 | Cellular |
| *I. kangtingensis* | E.D. Liu et al. 2662 (KUN) | Kangding, Sichuan, China | 1.61±0.15 | 1.16±0.15 | 1.39±0.06 | Cellular |
| *I. leucophyllus* | E.D. Liu et al. 3204 (KUN) | Jinchuan, Sichuan, China | 1.39±0.08 | 0.94±0.05 | 1.48±0.07 | Cellular |
| *I. loxothyrsus* | Y.P. Chen et al. EM259 (KUN) | Deqin, Yunnan, China | 1.5±0.2 | 1.02±0.1 | 1.48±0.16 | Cellular |
| *I. lungshengensis* | Y.P. Chen et al. EM084 (KUN) | Lingui, Guangxi, China | 1.18±0.07 | 1.01±0.08 | 1.18±0.07 | Cellular |
| *I. macrocalyx* | Y.P. Chen EM224 (KUN) | Hezhou, Guangxi, China | 1.91±0.1 | 1.61±0.16 | 1.19±0.08 | Glandular |
| *I. macrophyllus* | Y.P. Chen et al. EM034 (KUN) | Nanjing, Jiangsu, China | 1.91±0.11 | 1.42±0.1 | 1.35±0.08 | Cellular |
| *I. megathyrsus* | Y.P. Chen et al. SD359 (KUN) | Lijiang, Yunnan, China | 1.64±0.08 | 1.18±0.07 | 1.39±0.1 | Cellular |
| *I. nervosus* | Y.H. Zhang et al. s.n. (KUN) | Xinchang, Zhejiang, China | 1.65±0.09 | 1.16±0.07 | 1.43±0.1 | Glandular and pubescent |
| *I. oresbius* | H.J. Dong et al. D565 (KUN) | Lijiang, Yunnan, China | 1.69±0.04 | 0.94±0.08 | 1.81±0.15 | Cellular |
| *I. parvifolius* | E.D. Liu et al. 3096 (KUN) | Wenxian, Gansu, China | 1.44±0.17 | 1.06±0.15 | 1.37±0.07 | Cellular |
| *I. pharicus* | E.D. Liu et al. 3289 (KUN) | Damxung, Tibet, China | 1.64±0.08 | 1.25±0.09 | 1.32±0.09 | Cellular |
| *I. phyllostachys* | Y.P. Chen et al. EM146 (KUN) | Yongsheng, Yunnan, China | 1.67±0.1 | 1.13±0.07 | 1.48±0.08 | Cellular |
| *I. pleiophyllus* | H.J. Dong et al. D467 (KUN) | Lijiang, Yunnan, China | 1.66±0.04 | 1.05±0.03 | 1.59±0.05 | Cellular |
| *I. polystachys* | Y.P. Chen et al. EM140 (KUN) | Yongsheng, Yunnan, China | 1.33±0.08 | 0.86±0.02 | 1.54±0.09 | Cellular |
| *I. pseudoirroratus* | E.D. Liu et al. 2677 (KUN) | Daocheng, Sichuan, China | 2.12±0.09 | 1.33±0.1 | 1.59±0.13 | Cellular |
| *I. rosthornii* | Y.P. Chen EM127 (KUN) | Emeishan, Sichaun, China | 1.29±0.06 | 1.12±0.06 | 1.15±0.07 | Cellular |
| *I. rubescens* | Y.P. Chen et al. EM276 (KUN) | Guilin, Guangxi, China | 1.54±0.05 | 1.14±0.05 | 1.35±0.07 | Cellular |
| *I. rugosus* | G.X. Hu et al. 1209034 (KUN) | Gyaca, Tibet, China | 1.36±0.12 | 0.91±0.07 | 1.5±0.04 | Cellular |
| *I. scoparius* | E.D. Liu et al. 2710 (KUN) | Shangri-La, Yunnan, China | 1.44±0.05 | 0.99±0.06 | 1.46±0.07 | Cellular |
| *I. sculponeatus* | Y.P. Chen et al. XS-02 (KUN) | Kunming, Yunnan, China | 1.71±0.08 | 1.31±0.06 | 1.31±0.06 | Cellular |
| *I. serra* | Y.P. Chen et al. EM034 (KUN) | Nanjing, Jiangsu, China | 1.55±0.08 | 1.24±0.08 | 1.25±0.08 | Glandular and pubescent |
| *I. setchwanensis* | C.L. Xiang et al. 1239 (KUN) | Yanyuan, Sichuan, China | 1.31±0.09 | 1.02±0.09 | 1.29±0.12 | Cellular |
| *I. smithianus* | E.D. Liu et al.3186 (KUN) | Barkam, Sichuan, China | 1.47±0.09 | 1.15±0.06 | 1.29±0.06 | Cellular |
| *I. ternuifolius* | Y.P. Chen et al. EM256 (KUN) | Deqin, Yunnan, China | 1.43±0.08 | 0.95±0.05 | 1.5±0.07 | Glandular |
| *I. wardii* | G.X. Hu et al. 1209104 (KUN) | Milin, Tibet, China | 1.84±0.08 | 1.09±0.04 | 1.69±0.09 | Cellular |
| *I. weisiensis* | H.J. Dong et al. D623 (KUN) | Deqin, Yunnan, China | 1.85±0.09 | 1.3±0.06 | 1.42±0.07 | Cellular |
| *I. wikstroemioides* | E.D. Liu et al. 3336 (KUN) | Deqin, Yunnan, China | 1.52±0.04 | 1.03±0.06 | 1.48±0.08 | Cellular |
